# Emotional Responses to Non-Speech Sounds for Hearing-aid and Bimodal Cochlear-Implant Listeners

**DOI:** 10.1177/23312165221083091

**Published:** 2022-04-18

**Authors:** Marina M. Tawdrous, Kristen L. D'Onofrio, René Gifford, Erin M. Picou

**Affiliations:** 1 School of Communication Sciences and Disorders, 6221 Western University, 1151 Richmond St, London, ON, N6A 3K7; 2 Department of Hearing and Speech Sciences, Graduate School, 5718 Vanderbilt University, 1215 21st Ave South, Room 8310, Nashville, TN, 37232; 3 Department of Hearing and Speech Sciences, School of Medicine, 12328 Vanderbilt University Medical Center, 1215 21st Ave South, Room 8310, Nashville, TN, 37232

**Keywords:** emotion, cochlear implant, hearing aid, valence, arousal, hearing loss, affect

## Abstract

The purpose of this project was to evaluate differences between groups and device configurations for emotional responses to non-speech sounds. Three groups of adults participated: 1) listeners with normal hearing with no history of device use, 2) hearing aid candidates with or without hearing aid experience, and 3) bimodal cochlear-implant listeners with at least 6 months of implant use. Participants (*n* = 18 in each group) rated valence and arousal of pleasant, neutral, and unpleasant non-speech sounds. Listeners with normal hearing rated sounds without hearing devices. Hearing aid candidates rated sounds while using one or two hearing aids. Bimodal cochlear-implant listeners rated sounds while using a hearing aid alone, a cochlear implant alone, or the hearing aid and cochlear implant simultaneously. Analysis revealed significant differences between groups in ratings of pleasant and unpleasant stimuli; ratings from hearing aid candidates and bimodal cochlear-implant listeners were less extreme (less pleasant and less unpleasant) than were ratings from listeners with normal hearing. Hearing aid candidates’ ratings were similar with one and two hearing aids. Bimodal cochlear-implant listeners’ ratings of valence were higher (more pleasant) in the configuration without a hearing aid (implant only) than in the two configurations with a hearing aid (alone or with an implant). These data support the need for further investigation into hearing device optimization to improve emotional responses to non-speech sounds for adults with hearing loss.

## Introduction

Permanent, bilateral hearing loss is associated with psychosocial consequences, such as reduced quality of life (Dalton et al., 2003), increased depressive symptoms (Kramer et al., 2002), and increased isolation (Hawthorne, 2008). These inter-related psychosocial consequences of hearing loss might be partly attributable to reduced audibility and difficulty understanding speech, especially in noise (Humes & Roberts, 1990; Peters et al., 1998; Plomp, 1976). However, everyday listening and communication experiences are not strictly focused on speech perception. The perception and recognition of nonlinguistic, affective information is important for social communication (Kiss & Ennis, 2001; Zajonc, 1980) and will be referred to hereafter as ‘emotion recognition.’ Emotion recognition tasks typically involve participant judgement of the emotion portrayed (e.g., categorical judgement) and can be accomplished with speech (vocal emotion recognition; e.g., Most & Aviner, 2009) or music (musical emotion recognition; e.g., Ambert-Dahan et al., 2015). The acoustic cues important for vocal emotion recognition include mean fundamental frequency (F0), overall level, and F0 variability (e.g., Banse & Scherer, 1996; Paulmann et al., 2008). For example, anger and elation have high mean F0 and high level (Paulmann et al., 2008; Pell et al., 2009), whereas sadness exhibits low level, low mean F0, and little F0 variability (Juslin & Laukka, 2003). For music, emotion is conveyed through mode (e.g., major vs. minor) and tempo (e.g., fast vs. slow; Eerola & Vuoskoski, 2013), where pleasant songs are more likely to be in a major mode and faster in tempo than unpleasant or sad ones (Gosselin et al., 2005; Peretz et al., 1998).

Adults who have hearing loss demonstrate deficits on emotion recognition tasks. For example, adults who have bilateral, mild to moderately-severe sensorineural hearing loss, traditionally considered hearing aid (HA) candidates, demonstrate poorer performance on tasks of vocal emotion recognition compared to their peers with better hearing (Christensen et al., 2019; Singh et al., 2019). These deficits in bilateral HA users might be attributable to loss of low-frequency audibility, as performance on these tasks is correlated with low-frequency audiometric thresholds (e.g., below ∼500 Hz; Rigo & Lieberman, 1989; Singh et al., 2019). There is also clear evidence that cochlear implant (CI) users demonstrate emotion recognition deficits for both speech and music stimuli (Caldwell et al., 2015; Chatterjee et al., 2015; Damm et al., 2019; Deroche et al., 2019; D’Onofrio et al., 2020; Jiam et al., 2017; Luo et al., 2007; Shirvani et al., 2014). Such deficits have largely been attributed to the limitations of envelope-based signal processing, which prevent sufficient spectro-temporal detail in the CI-mediated signal (Chatterjee & Peng, 2008; Hsiao & Gfeller, 2012; Jiam et al., 2017; Luo et al., 2007).

### Emotional Responses to Sounds

Recognition of emotion in speech and music is not the only way emotion perception is important for typical functioning. An individual's responses to potentially emotional events (e.g., bees buzzing, crying, music) have pervasive impacts that can be measured in a variety of domains, but the effects are asymmetric. Aversive or unpleasant stimuli prepare a body to respond to negative events (Taylor, 1991), facilitate focused attention (Baumeister et al., 2001; Kensinger, 2009), and even improve speech recognition (Dupuis & Pichora-Fuller, 2014). Conversely, pleasant stimuli motivate people to approach an event and broaden attention (Bradley et al., 2001; Fredrickson & Branigan, 2005), with positive effects on stress recovery (Alvarsson et al., 2010; Sandstrom & Russo, 2010) and creative thinking (Fredrickson, 2001).

The dimensional view of emotion provides a convenient framework for measuring the extent to which an individual is affected by stimuli by having them rate their response to the stimulus along a combination of two or more dimensions (e.g., Faith & Thayer, 2001; Osgood et al., 1957). Among the available dimensions, valence and arousal often account for most of the variability in emotion (Bradley & Lang, 1994), where valence indicates the hedonistic value (pleasant / unpleasant) and arousal indicates the intensity of the emotion (exciting / calming). Acoustically, level is a robust cue for arousal; higher level speech and music are perceived as more exciting (Goudbeek & Scherer, 2010; Ilie & Thompson, 2006; Laukka et al., 2005; Schmidt, Herzog, et al., 2016). The acoustic cues supporting ratings of valence are less clear than those for arousal (Goudbeek & Scherer, 2010; Laukka et al., 2005) and depend on stimulus type. For example, high-pitched speech and low-pitched music are both associated with lower ratings of valence than low-pitched speech and high-pitched music (Ilie & Thompson, 2006; Schmidt, Herzog, et al., 2016; Schmidt, Janse, et al., 2016). Similarly, high ratings of pleasantness are elicited by loud music and quiet speech (Weninger et al., 2013).

Using ratings of valence and arousal (rather than categorical judgments of emotion), evidence suggests CI users demonstrate reduced ratings of arousal compared to their peers with normal hearing (Ambert-Dahan et al., 2015; Paquette et al., 2018). Although some investigators report ratings of valence might not be different between CI users and adults with normal hearing (Ambert-Dahan et al., 2015; Rosslau et al., 2012), there is also some evidence to suggest CI-users rate speech or music as less extreme (less pleasant and less unpleasant) than their peers with normal hearing (Caldwell et al., 2015; D’Onofrio et al., 2020; Paquette et al., 2018). Furthermore, the acoustic cues that CI users rely on for valence ratings are different than those listeners with normal hearing primarily use, especially in music; CI users rely more heavily on tempo than on spectral information (Caldwell et al., 2015; D’Onofrio et al., 2020).

Compared to music and speech, relatively less is known about the effects of hearing loss on emotional responses to non-speech sounds, especially sounds that are commonly encountered (e.g., birds chirping, glass breaking). For non-speech sounds, level is also a robust cue for arousal (Buono et al., 2021; Ma et al., 2012), although the acoustic cues that carry valence of non-speech sounds are less clear than those for speech or music. For example, despite its role in emotion perception of speech and music, F0 has not been related to ratings of valence of non-speech sounds (Picou, 2016; Weninger et al., 2013). However, changes in spectral content of signals have been related to changes in ratings of valence; stimuli with more limited bandwidths have been shown to elicit lower ratings of valence than the same sounds presented with full bandwidth (Buono et al., 2021; Ma & Thompson, 2015).

As with speech and music, emerging work suggests people with hearing loss demonstrate different emotional responses to non-speech sounds than their peers with normal hearing (Husain et al., 2014; Picou & Buono, 2018). For example, Picou (2016) evaluated ratings of valence and arousal in response to non-speech sounds for similarly aged listeners with normal hearing (NH) and mild- to moderately-severe bilateral sensorineural hearing loss. Results indicated that participants with hearing loss exhibited valence responses that were less extreme (less pleasant and less unpleasant) than their peers’.

To our knowledge, ratings of valence and arousal in response to non-speech sounds for bimodal CI listeners have not been reported, nor have direct comparisons between adults with normal hearing, adults who are HA candidates, and adults who are CI users. Given the work in other areas of emotion perception, is expected that the effects of hearing loss on emotional responses to these everyday non-speech sounds will be quite different for adults who are HA candidates (with normal/mild sloping to moderate/severe sensorineural hearing loss) than for adults who use a HA in conjunction with a cochlear implant (CI) in the opposite ear (bimodal CI configuration). Moreover, given the potential for level and spectral cues to influence ratings of arousal and valence, it is also likely assistive hearing device configuration might affect emotion perception for listeners with hearing loss.

### Assistive Hearing Device Configurations

It is not clear how to optimize assistive hearing device configurations for emotion perception. For hearing aid candidates, HAs can improve audibility and consequently speech recognition (Alcántara et al., 2003; Humes et al., 2002; Picou et al., 2013). However, no investigators have reported that the addition of hearing aids improves emotion recognition performance (Goy et al., 2018; Singh et al., 2019) or ratings of valence of speech (Schmidt, Herzog, et al., 2016). Similarly, Picou, Rakita, et al. (2021) reported no significant benefit of HA use on emotional responses to non-speech sounds. Instead, HAs reduced ratings of valence in response to all categories of sounds (pleasant, neutral, unpleasant). Thus, although HAs improve audibility of sounds and would be expected to improve ratings of valence, the improvement in audibility might be offset by the increased loudness of sounds with hearing aids; loud sounds have been shown to result in low ratings of valence, even if the sounds are expected to be pleasant (Atias et al., 2019; Picou, 2016; Picou, Rakita, et al., 2021).

Clinically, bilateral hearing aids are generally recommended for people with symmetrical hearing loss (for review of current hearing aid fitting standard, see Picou, Roberts, et al., 2021), yet patients’ preferences for bilateral hearing aids can be variable, with estimates of preference ranging from ∼90% (Boymans et al., 2008; Erdman & Sedge, 1981) to only ∼30% (Erdman & Sedge, 1981; Schreurs & Olsen, 1985; Vaughan-Jones et al., 1993). It is possible that one of the reasons patients might prefer a single HA over bilateral HAs, despite clear benefits for bilateral HAs on laboratory-based speech recognition tasks (Boymans et al., 2008; Freyaldenhoven et al., 2006; Hawkins & Yacullo, 1984; Köbler et al., 2001; Ricketts et al., 2019), is related to differences in emotion perception with unilateral or bilateral hearing aids. Thus, it is important to identify if there are differences in the emotional responses to sounds for people who are wearing one or two HAs.

For CI users, the challenges of CI-mediated listening could be mitigated in some cases via the combined use of acoustic and electric stimulation. With the expansion of CI candidacy criteria in recent years, an increasing number of patients now have useable, residual hearing. Indeed, approximately 60–72% of adult CI recipients have some degree of acoustic hearing in the non-CI ear, and are thus, candidates for bimodal stimulation (Dorman & Gifford, 2010; Holder et al., 2018). Significant benefit from the addition of acoustic hearing has been shown for speech recognition (e.g., Dunn et al., 2005; Gifford et al., 2018; Gifford & Dorman, 2019; Potts et al., 2009; Sladen et al., 2018), perception of suprasegmental features of speech (Davidson et al., 2019; Most, Harel, et al., 2011), music perception (Cheng et al., 2018; Crew et al., 2015; Cullington & Zeng, 2011; Dorman et al., 2008; El Fata et al., 2009; Kong et al., 2005, 2012; Plant & Babic, 2016; Sucher & McDermott, 2009), emotion recognition of speech sounds (Most, Gaon-Sivan, et al., 2011), musical sound quality (D'Onofrio & Gifford, 2021), and musical emotion perception (D’Onofrio et al., 2020; Giannantonio et al., 2015; Shirvani et al., 2016). The bimodal benefit evidenced in the aforementioned studies - that is, the improved performance achieved with the contribution of acoustic hearing (via HA) in the contralateral ear – is largely the result of increased access to features poorly transmitted via the CI, specifically fundamental frequency (F0; e.g., Gifford et al., 2021; Kong et al., 2004, 2005) and temporal fine structure (e.g., Kong & Carlyon, 2007; Sheffield & Gifford, 2014). However, it is not clear if bimodal CI benefits extend to emotional responses to non-speech sounds for CI users.

### Purpose

The purpose of this project was two-fold: 1) to evaluate the between-group differences in emotional responses to non-speech sounds between listeners with normal hearing, hearing aid candidates, and bimodal CI listeners and 2) to evaluate the effects of device configuration on emotional responses to non-speech sounds. To evaluate the effects of group membership, three groups of listeners were tested with a standard-of-care intervention (no device, bilateral HAs, or bimodal CI configuration). It was expected that, relative to their peers with NH, both groups would demonstrate ratings of valence that were less extreme (less pleasant and less unpleasant), even while using assistive hearing device technology, due to the continued difficulties with emotion perception adults exhibit with hearing aids (e.g., Goy et al., 2018; Picou, Rakita, et al., 2021; Singh et al., 2019) and cochlear implants (Caldwell et al., 2015; D’Onofrio et al., 2020; Jiam et al., 2017). Furthermore, based on the noted reduced range with increasing pure-tone average (Picou & Buono, 2018), it was expected that bimodal CI listeners would demonstrate larger deficits (smaller range of emotional responses) than HA candidates, who typically have lesser degrees of hearing loss.

The second purpose was to evaluate the effect of device configuration. For HA candidates, the configuration options were unilateral or bilateral HA fitting. Ideally, the range of emotional responses would be broadest under bilateral HA conditions, given the current clinical recommendations for bilateral fittings in most cases (e.g., Picou, Roberts, et al., 2021). For bimodal CI listeners, it was predicted that emotional responses would be most similar to those of listeners with NH in the bimodal configuration (CI and contralateral HA) relative to HA- or CI- only conditions, given the work demonstrating the benefits of a contralateral HA for emotion perception of music (e.g., D’Onofrio et al., 2020; Giannantonio et al., 2015) and speech (e.g., Most, Gaon-Sivan, et al., 2011).

## Methods

### Participants

**Figure 1.**
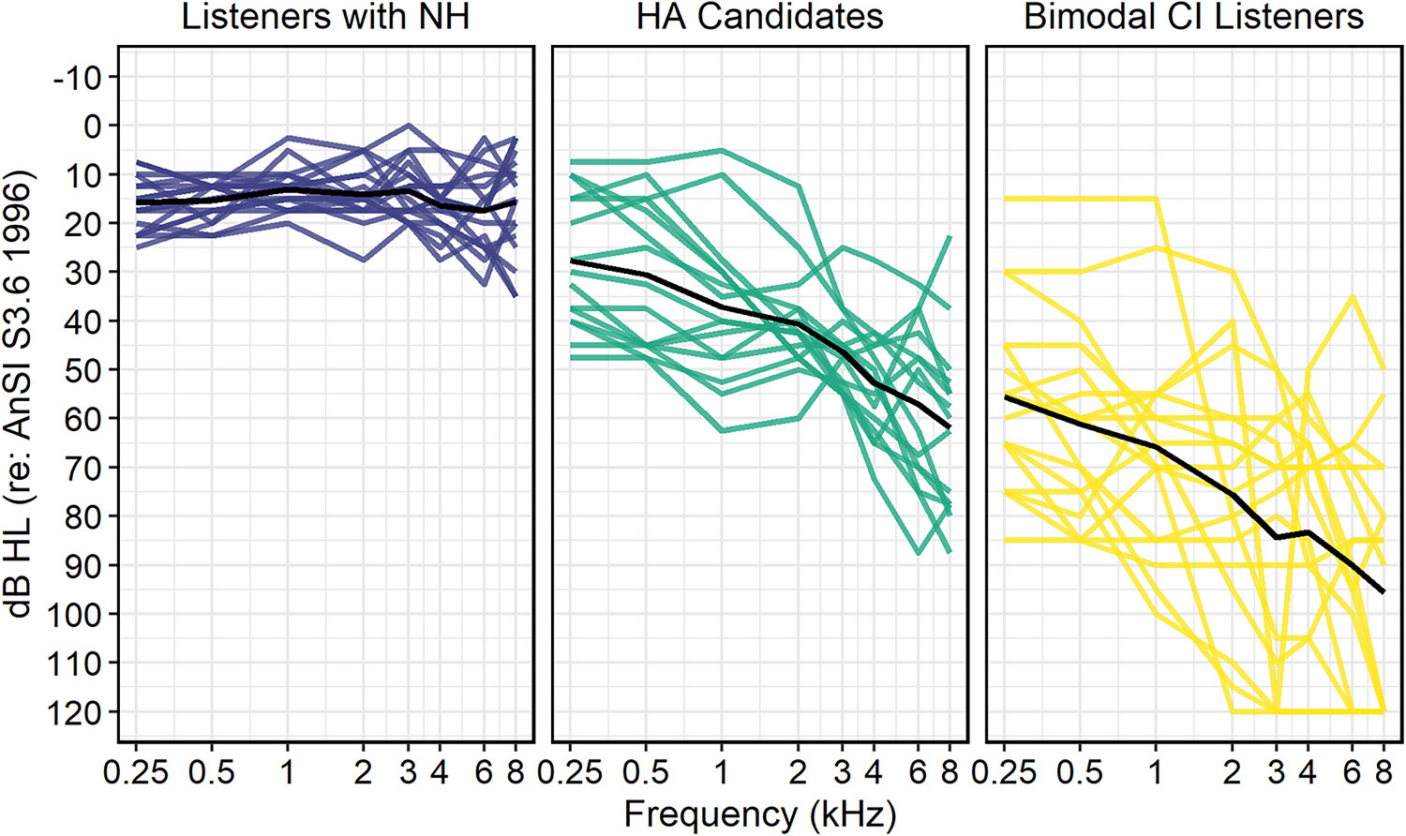
Audiometric thresholds for the NH, HA, and bimodal groups. Group mean thresholds are shown in black. For listeners with normal hearing and hearing aid candidates, mean right and left ear thresholds are displayed. For bimodal CI listeners, thresholds for the non-implanted ear are displayed.

Participants were recruited through review of clinic records in the Department of Audiology at Vanderbilt University Medical Center and through mass e-mail solicitation to the Vanderbilt University Medical Center community. Three groups of 18 adults participated: 1) listeners with NH, 2) HA candidates, and 3) bimodal CI listeners. Table 1 displays demographic information and Figure 1 displays pure-tone air conduction thresholds for the three groups. All participants denied neurogenic disorders, pharmacologic treatment for mood disorders, or cognitive decline. All participants demonstrated low risk of clinical depression, as assessed using the Hospital Anxiety and Depression Scale (Zigmond & Snaith, 1983). Table 1 reveals the participant groups were matched on the measures of anxiety, depression, and perceived ability to recognize vocal emotion, yet they differed based on degree of hearing loss, hearing aid experience, and duration of hearing loss. In addition, the groups differed slightly in age and gender, where HA candidates were approximately 9 years older than the other two groups and there were more females in the group of NH listeners than in the two groups of participants with hearing loss. Detailed demographic data for all participants are displayed in Appendix A (listeners with NH), Appendix B (HA candidates), and Appendix C (bimodal CI listeners). Testing was conducted with approval from the Institutional Review Board at Vanderbilt University Medical Center. Participants were compensated for their time at an hourly rate.

**Table 1.** Participant Demographics for the Three Groups of Listeners *(*n = 18 in Each Group).

Characteristic		Normal Hearing, *N* = 17*^1^*	Hearing Aid Candidates, *N* = 15*^1^*	Bimodal Listeners, *N* = 17*^1^*	p-value*^2^*
Age	Years	55.18 (10.01)	64.00 (5.26)	55.41 (18.52)	0.012
Gender	Female	14 (82%)	7 (47%)	7 (41%)	0.032
	Male	3 (18%)	8 (53%)	10 (59%)	
PTA	dB HL	13.06 (3.54)	38.47 (10.32)	71.53 (17.00)	<0.001
Duration of hearing loss	Years		11.73 (14.34)	22.15 (16.94)	0.029
HADS-A	Score	6.12 (2.91)	5.53 (3.76)	6.00 (4.47)	0.83
HADS-A	Score	1.76 (1.48)	3.47 (3.34)	3.93 (2.76)	0.10
EmoCheq	Score	35.06 (15.11)	39.93 (17.17)	43.93 (13.68)	0.17
Devices	None	17 (100%)	4 (27%)	0 (0%)	<0.001
	One	0 (0%)	1 (6.7%)	0 (0%)	
	Two	0 (0%)	10 (67%)	17 (100%)	
Hearing aid experience	Yes		11 (73%)	17 (100%)	0.038
Hearing aid use	Years		6.15 (10.34)	13.84 (9.80)	0.028
Cochlear implant use	Years			3.42 (3.52)	
Cochlear implant	Cochlear			8 (47%)	
	Advanced Bionics			9 (53%)	

*^1^* Mean (SD); n (%), *^2^* Kruskal-Wallis rank sum test; Pearson's Chi-squared test; Fisher's exact test, *Note:* PTA = better ear, pure-tone average (0.5, 1, 2, 4 kHz); HADS = Hospital Anxiety and Depression Scale.

### Hearing Aid Fitting

#### Hearing Aid Candidates

For the purpose of this study, users were fit with research HAs (behind-the-ear, Phonak Ambra V90). The HAs were coupled using foam, non-custom eartips (Comply^TM^), which resulted in occluding fittings for most participants. The HAs were programed for each participant according to prescriptive targets from the National Acoustic Laboratories – Nonlinear v 2 (NAL-NL2; Keidser et al., 2012) for a bilateral fitting. Fittings were verified using recorded speech passages presented at 65 dB SPL and a probe-microphone verification system (Audioscan Verifit). One participant, a 70 year old male, was under fit by 7 dB at 4000 Hz in the right ear. Otherwise, all fittings were within 5 dB of NAL-NL2 prescriptive targets 250–4000 Hz. All advanced digital features were deactivated (digital noise reduction, wind reduction, speech enhancement, frequency lowering), except feedback reduction, which was personalized for each participant. The HA microphone was set to be mildly directional, with an average directivity index designed to overcome the microphone location effects of a behind-the-ear instrument. Participants in the HA group completed testing described below with two HAs (bilateral condition) and one HA (unilateral condition). In the unilateral condition, one HA was removed and that ear was unoccluded; the left ear was the test ear for half of the participants.

#### Bimodal CI Listeners

Bimodal CI listeners were also fitted with a behind-the-ear research HA for the purpose of the study (Phonak Bolero V90-SP). The HA, fitted to the non-implanted ear, was coupled with a custom, fully-occluding earmold made for the purpose of this study, or with the participant's own, fully-occluding earmold if they used a custom mold regularly. Consistent with the HA group, the advanced features on the HAs used for the bimodal group were deactivated, with the exception of feedback reduction. As with the HA group, the microphone was set to be mildly directional. HA gain was programmed and verified to match NAL-NL2 targets. Match to target was within 10 dB 250–4000 Hz for 10 participants. For 8 participants, adequate gain could not be achieved for 4000 Hz and real ear aided responses were more than 10 dB below NAL-NL2 targets.

The bimodal CI listeners also used a CI. The CI map was not adjusted for this study; their existing maps were used. Participants either used an Advanced Bionics (*n* = 9) or a Cochlear (*n* = 9) implant. In all cases, their ‘Every day’ program was used during testing. Prior to testing, CI-aided thresholds were completed in the sound field to warbled pure tones. Thresholds were in the range of 20–30 dB HL from 250–6000 Hz for most qualifying participants. Three participants demonstrated thresholds up to 40 dB HL.

### Stimuli

Participants provided ratings of arousal and valence using the Self-Assessment Manikin (SAM; Bradley & Lang, 1994). The SAM is a non-verbal, pictorial tool for measuring emotion along the dimensions of valence, arousal, and dominance. For each dimension, the SAM includes 5 cartoon figures representing the range of emotions along the dimension (e.g., smiling to frowning) and participants make their ratings on a range of 1 to 9 based on numbers equally spaced under the 5 figures. For this study, only the valence and arousal dimensions were used. For both dimensions, the captions “how pleasant / unpleasant do you feel” and “how excited or calm do you feel” were placed above the pictures for valence and arousal, respectively.

Participants rated valence and arousal in response to non-speech sounds from the International Affective Digitized Sounds Corpus (IADS; Bradley & Lang, 2007). The corpus includes 167 non-speech examples of animal noises (e.g., cows mooing), human social noises (e.g., laughter), bodily noises (e.g., belching), environmental sounds (e.g., office noises), and music (e.g., acoustic guitars). Bradley and Lang (2007) published ratings of valence, arousal, and dominance elicited from college students with presumably NH. Of these 167 tokens, 75 were used in this study. The 75 tokens were the same ones used by our previous studies (Picou, 2016; Picou & Buono, 2018; Picou, Rakita, et al., 2021). The tokens were modified from their original format in two ways. First, their duration was shortened from 6 s to 1.5 s by selecting a representative sample of the token. Second, their levels were normalized so they all had the same peak level (-3.01 dB relative to the soundcard maximum). Both modifications were made using Adobe Audition (v CSS5). Based on the ratings provided by listeners with NH in a previous study (Picou, 2016), the tokens were assigned to one of three categories, which varied based on their expected valence (pleasant, neutral, unpleasant). Categories and brief descriptions of all sounds are displayed in Appendix D. Sounds were presented at 65 dB SPL.

### Procedures

Prior to testing, the level was calibrated using a steady-state signal with the same long-term average spectrum as the stimuli used during testing. A sound level meter (Amprobe SM-10) at the position of the participant's ear, without a participant in the room, was used to verify the level. Following informed consent, participants completed the Hospital Anxiety and Depression Scale and then underwent hearing evaluation (pure-tone, air conduction thresholds) and HA fitting in a quiet, clinic-like environment. Then, they rated valence and arousal in a sound-attenuating audiometric test booth. Testing was blocked; they rated valence and arousal in response to all 75 sounds in one condition before switching conditions or taking a break. Within the condition, sounds in all three categories were randomly presented. Participants with NH rated sounds in only one condition (unaided). Participants who were HA users rated sounds in two conditions (unilateral HA, bilateral HAs). Participants who were bimodal CI listeners rated sounds in three conditions (HA only, CI only, HA + CI). For participants who completed more than one condition, their condition order was counterbalanced. Breaks were provided as needed during testing.

### Test Environment

The participant was seated in the center of the audiometric booth (4.0 × 4.3 × 2.7 m) with a loudspeaker (Tannoy Series 600) placed 1.25 m in front of the participant. A computer monitor (21.5-in Dell S2240T) was placed directly below the loudspeaker and in front of the participant. During testing, the monitor displayed a small, black fixation cross on a white screen during sound presentation. Immediately after the sound finished, the SAM stimuli for rating valence were displayed (caption, five pictures, numbers from 1 to 9). A participant then selected their rating of valence using a keypad (USB; Targus). Then the SAM stimuli for rating arousal were displayed (caption, five pictures, numbers from 1 to 9) and participants provided a rating of arousal. When they were ready to advance, they would press ‘Enter’ and the next sound was presented. The experimental timing and data collection were controlled using Presentation (Neurobehavioral Systems v 14) on an experimental computer (Dell) outside the test booth. The computer monitor inside the test booth displayed a cloned image of the experimental computer monitor. From the experimental computer, which stored the stimuli for testing, the sounds were routed to an audiometer for level control (Madsen Orbiter 922 v.2), to an amplifier (Russound), and then to the loudspeaker.

### Data Analysis

Prior to analysis, five participants were excluded. A computer error prohibited responses from one participant with NH (54 year old female) from being recorded. In the group of HA users, two participants (72 year old female, 50 year old female) provided only one rating (valence or arousal) in all conditions rather than two ratings (valence and arousal). Also in the HA candidate group, one participant provided a single rating in one condition (bilateral HA; 70 year old female). All three participants had no HA experience. In addition, one participant in the bimodal listener group did not have data recorded due to experimenter error in the “HA only” condition. Therefore, these participants were excluded from further analysis.

Scores for individual participants were calculated by taking the average rating of valence or arousal in each stimulus category for each condition. Separate linear effects models were constructed to address each of the research questions regarding 1) hearing loss and 2) device configuration. To examine the effect of the hearing loss on emotion, the models of valence and arousal included a single between-group factor (NH, HA candidate, bimodal CI listener) and one within-participant variable (stimulus category; pleasant, neutral, unpleasant). Prior to analysis, the ratings of valence and arousal were z-score transformed for each participant in the “maximum” device configuration (no hearing device for listeners with NH, the bilateral HA condition for HA users, and the bimodal condition (CI + HA) for the bimodal CI listeners). To examine the effect of device configuration on emotional responses, the models of valence and arousal included two within-participant factors, stimulus category and device configuration. Separate linear mixed effects models were constructed for the HA candidates (unilateral or bilateral HAs) and bimodal CI listeners (HA only, CI only, CI + HA), each with participant as a random factor. Analysis of variance (ANOVA) was conducted on each linear model; significant main effects and interactions were explored using pairwise comparisons of the estimated marginal means using Satterthwaite degrees of freedom and false discovery rate correction (Benjamini & Hochberg, 1995).

All analyses were completed within R (v 4.1.0; R Core Team, 2021), where the linear mixed-effect models were constructed using the **lme4** package (Bates et al., 2015), the ANOVAs were done using the **stats** package from base R, and the estimated marginal means with the pairwise comparisons were done using the **emmeans** package (Lenth, 2019).

## Results

### Differences Between Groups

**Figure 2.**
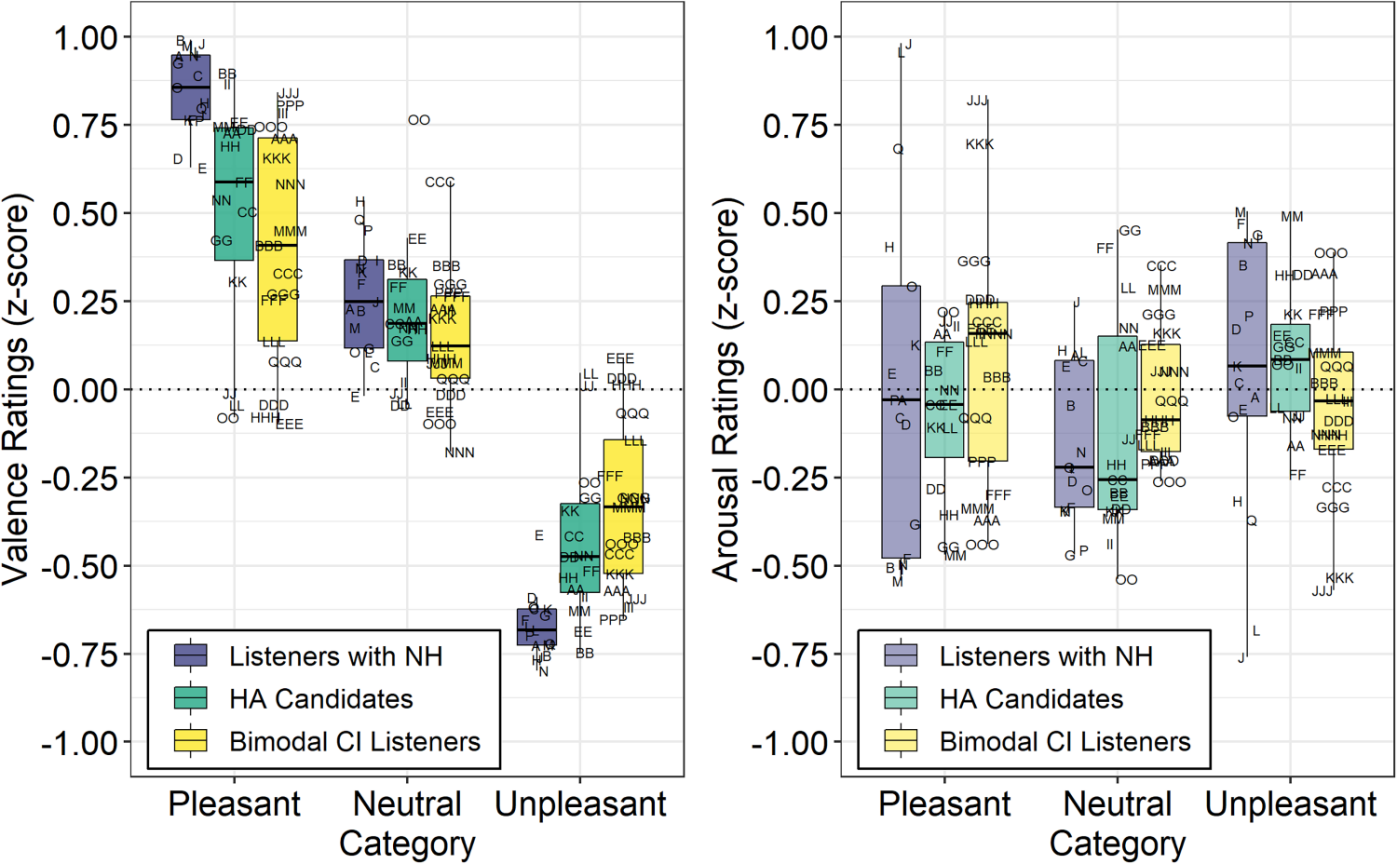
Normalized ratings of valence (left panel) and arousal (right panel) for listeners with NH, HA candidates, and bimodal CI listeners. See Appendices A-C for listeners’ identification code and demographics details.

Transformed ratings of valence and arousal for the listeners with NH, HA candidates, and bimodal CI listeners are displayed in Figure 2 (left panel). Analysis of z-score transformed ratings of valence revealed significant contributions of Category (*F* [2, 3616] = 575.354, *p* < 0.001) and a significant Group × Category interaction (*F* [4, 3616] = 27.993, *p* < 0.001). The effect of Group alone was not significant (*F* [2, 3616] < 1.0, *p* = 1.00). As a result of the significant Group × Category interaction, the estimated marginal means were calculated on the full model to evaluate the effect of group membership for each category separately. The results, displayed in Table 2, reveal significant differences between groups, in response to pleasant and unpleasant stimuli. Specifically, ratings from HA candidates and bimodal CI listeners were less extreme (less pleasant, less unpleasant) compared to listeners with NH. There were no significant effects of group membership in the neutral stimuli category.

**Table 2.** Pairwise Comparisons of Ratings of Valence (z-Score).

Stimulus Category	Group Contrast	Estimate	Standard Error	z ratio	*p*
Pleasant	NH - HA Candidate	0.34	0.06	5.43	<0.0001***
Pleasant	NH - Bimodal Listener	0.44	0.06	7.31	<0.0001***
Pleasant	HA Candidate – Bimodal CI Listener	0.10	0.06	1.66	0.096
Neutral	NH - HA Candidate	0.05	0.08	0.62	0.533
Neutral	NH - Bimodal Listener	0.11	0.08	1.50	0.403
Neutral	HA Candidate - Bimodal Listener	0.07	0.08	0.82	0.533
Unpleasant	NH - HA Candidate	−0.24	0.05	−4.75	<0.0001***
Unpleasant	NH - Bimodal Listener	−0.34	0.05	−7.01	<0.0001***
Unpleasant	HA Candidate - Bimodal Listener	−0.10	0.05	−2.03	0.043*

Note: NH = normal hearing; HA = hearing aid; * indicates *p* < 0.05, ** indicates *p* < 0.01, *** indicates *p* < 0.001.

Analysis of ratings of arousal revealed significant contribution of Category (*F* [2, 3616] = 4.02, *p* < 0.05) and a significant Group × Category interaction (*F* [4, 3616] = 3.39, *p* < 0.01). The effect of Group alone was not significant (*F* [2, 3616 < 1.0, *p* = 1.00). Differences between groups were small and variable (see Figure 2, right panel). Follow-up pairwise comparisons, displayed in Table 3, revealed only one of the differences between groups survived adjustment for family-wise error rate. Bimodal CI listeners rated unpleasant sounds as less arousing than did HA candidates. These data indicate ratings of arousal were generally not different between groups.

**Table 3.** Pairwise Comparisons of Ratings of Arousal (z-Score).

Stimulus Category	Group Contrast	Estimate	Standard Error	z ratio	*p*
Pleasant	NH - HA Candidate	0.08	0.07	1.12	0.297
Pleasant	NH - Bimodal Listener	−0.07	0.07	−1.04	0.297
Pleasant	HA Candidate - Bimodal Listener	−0.15	0.07	−2.12	0.102
Neutral	NH - HA Candidate	−0.02	0.09	−0.21	0.830
Neutral	NH - Bimodal Listener	−0.13	0.09	−1.45	0.350
Neutral	HA Candidate - Bimodal Listener	−0.11	0.09	−1.19	0.350
Unpleasant	NH - HA Candidate	−0.05	0.06	−0.78	0.436
Unpleasant	NH - Bimodal Listener	0.10	0.06	1.78	0.113
Unpleasant	HA Candidate - Bimodal Listener	0.15	0.06	2.49	0.038*

Note: NH = normal hearing; HA = hearing aid.

### Effect of Device Configuration

#### Hearing Aid Candidates

**Figure 3.**
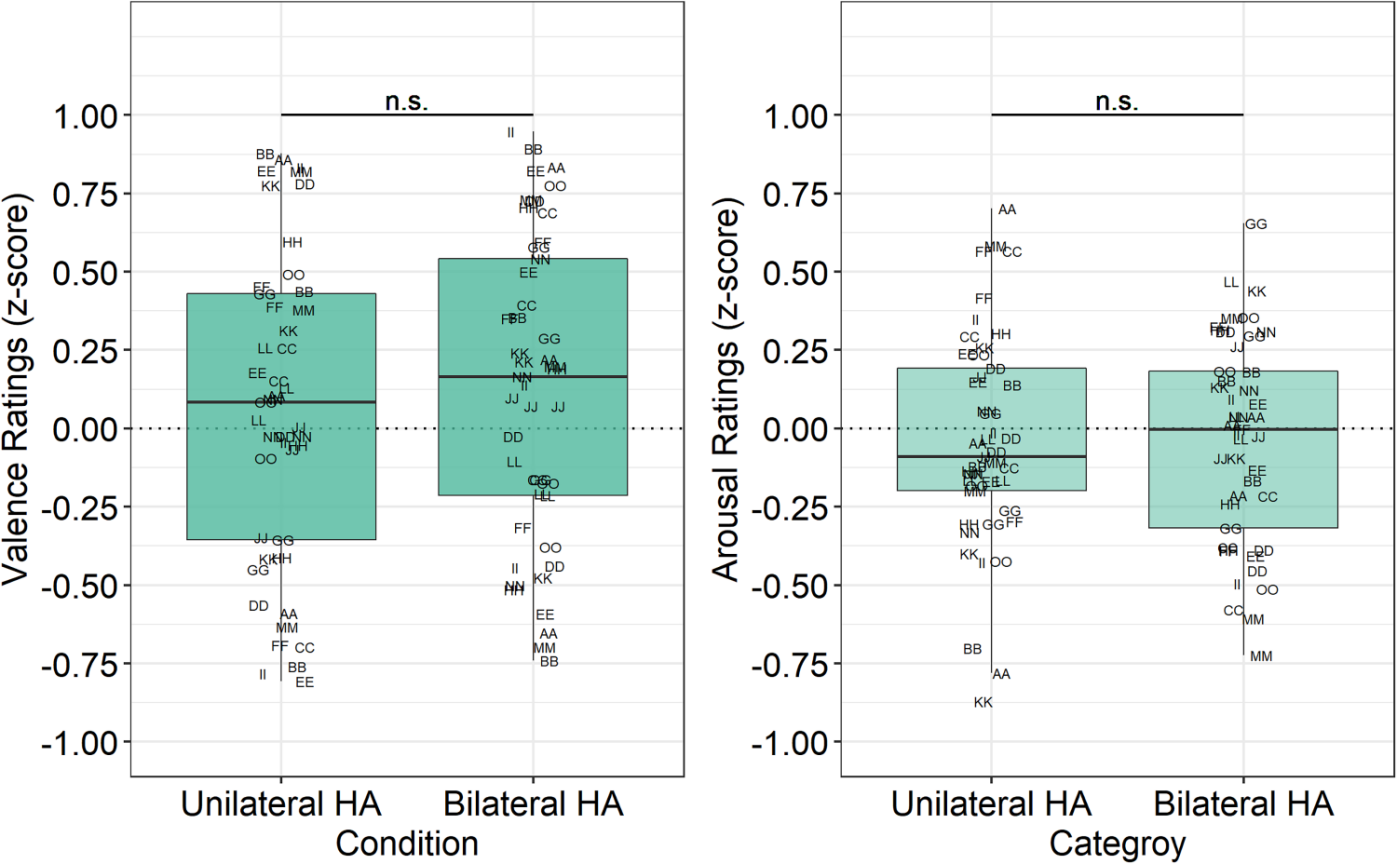
Normalized ratings of valence (left panel) and arousal (right panel) for listeners who are HA candidates. See Appendix B for participant identification code and demographics details.

Normalized, z-scored ratings of valence and arousal for the group of HA candidates are displayed in Figure 3. Analysis of ratings of valence revealed only a significant main effect of Category (*F* [2, 2208] = 236.18, *p* < 0.001). The effect of Configuration (*F* [1, 2208] = 1.66, *p* = 0.198) and the Configuration × Category interaction (*F* [2, 2208] = 0.19, *p* = 0.828) were not significant. As expected, ratings in response to pleasant stimuli were higher than in response to neutral stimuli (*M* difference = 0.77 points, *p* < 0.0001) or unpleasant stimuli (*M* difference = 2.00, *p* < 0.0001). In addition, ratings were lower in response to unpleasant sounds than neutral ones (*M* difference = 1.23, *p* < 0.0001). However, these results demonstrate that ratings were similar with unilateral and bilateral HAs (*M* rating difference = −0.11, *p* = 0.199).

Analysis of ratings of arousal also revealed only a significant main effect of Category (*F* [2, 2208] = 16.68, *p* < 0.001) and non-significant effects of Condition (*F* [2, 2208] = 0.79, *p* = 0.3738) and Category × Condition (*F* [2, 2208] = 0.34, *p* = 0.7125). Pairwise comparison testing revealed ratings of arousal were higher in response to the unpleasant stimuli than in response to the neutral stimuli (*M* difference = 0.44, *p* < 0.0001) or the pleasant stimuli (*M* difference = 0.37, *p* < 0.0001), whereas ratings were not different in response to neutral and pleasant stimuli (*M* difference = 0.72, *p* = 0.4712). Ratings of arousal were similar with unilateral and bilateral HA fittings (*M* rating difference = 0.04, *p* = 0.5754).

#### Bimodal CI Listeners

**Figure 4.**
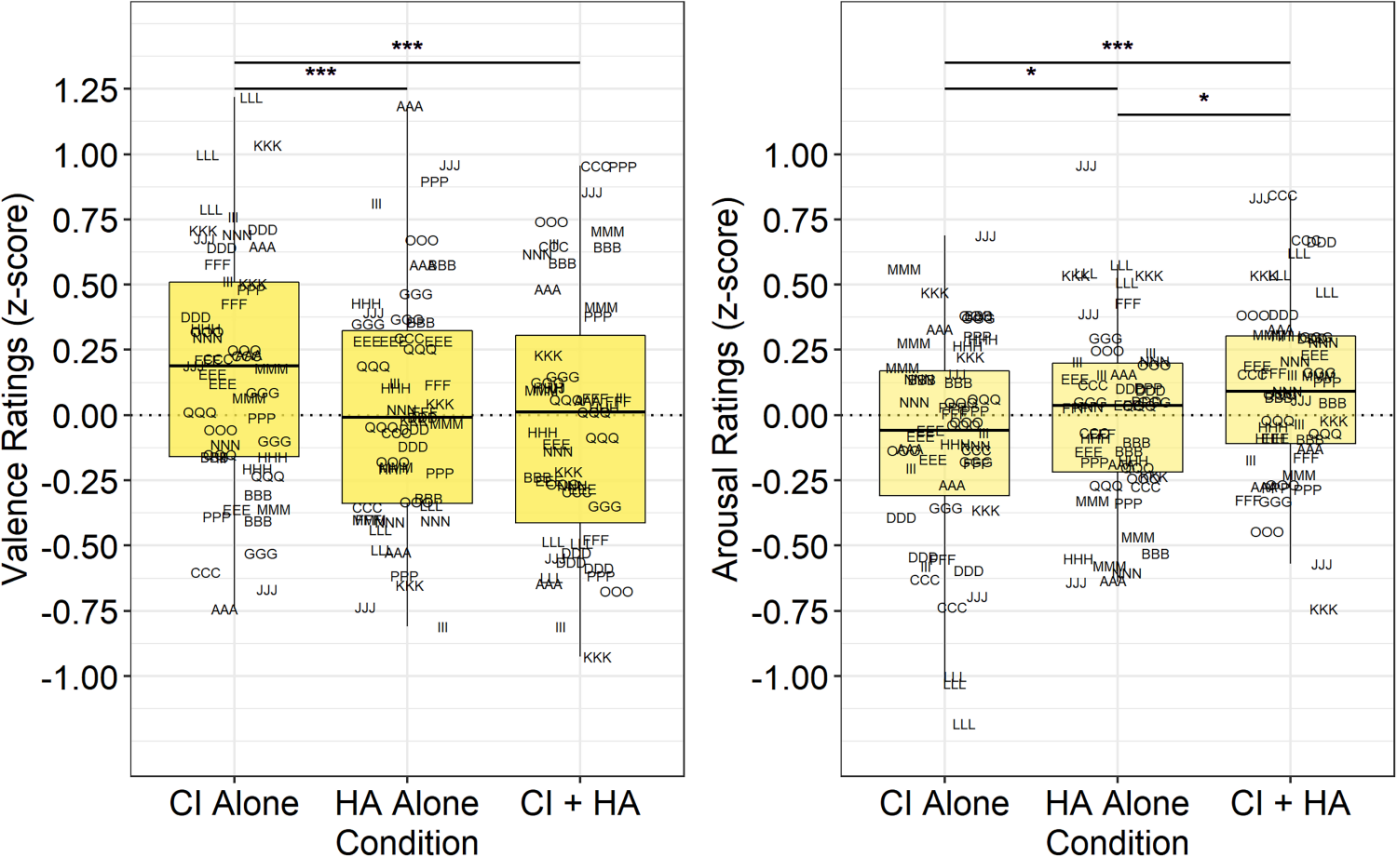
Normalized ratings of valence (left panel) and arousal (right panel) for bimodal CI listeners. See Appendix C for participant identification code and demographics details.

Normalized, z-scored ratings of valence and arousal for the group of bimodal CI listeners are displayed in Figure 4. Analysis of ratings of valence revealed significant effects of Configuration (*F* [2, 3774] = 13.81, *p* < 0.001) and Category (*F* [2, 3774] = 184.82, *p* < 0.001), but no significant Configuration × Category interaction (*F* [4, 3774] = 1.57, *p* = 0.179). Follow-up pairwise comparison testing revealed that all categories were significantly different from each other (*p* < 0.001). In addition, as displayed in Table 4 (top rows), ratings of valence were higher (more pleasant) with the CI alone relative to both the HA alone and CI + HA conditions.

**Table 4.** Pairwise Comparisons to Evaluate the Effect of Listening Configuration for Bimodal Listeners. Comparisons are Displayed for Ratings of Valence (top) and Ratings of Arousal (Bottom).

Rating	Contrast	Estimate	Standard Error	z ratio	*p*
Valence	CI + HA - CI alone	−0.18	0.04	−4.55	<.0001***
	CI + HA - HA alone	−0.04	0.04	−0.99	.324
	CI alone - HA alone	0.14	0.04	3.57	<.001***
Arousal	CI + HA - CI alone	0.20	0.04	4.66	.021*
	CI + HA - HA alone	0.10	0.04	2.30	<.0001***
	CI alone - HA alone	−0.10	0.04	−2.36	.021*

*Note:* CI = cochlear implant; HA = hearing aid; *** indicates *p* < 0.001.

Analysis of ratings of arousal revealed significant main effects of Configuration (*F* [2, 3774] = 9.717, *p* < 0.001) and Category (*F* [2, 3774] = 4.201, *p* < 0.05), but no significant Configuration × Category interaction (*F* [4, 3774] = 1.083, *p* = 0.363). Follow-up pairwise comparison testing revealed that ratings of arousal were higher in response to pleasant stimuli than with neutral (*M* difference = 0.11, *p* < 0.05) or unpleasant (*M* difference = 0.10, *p* < 0.05) stimuli. Ratings of arousal were not different in response to neutral or unpleasant stimuli (*M* difference = 0.001, *p* = 0.819). In addition, as displayed in Table 4 (bottom rows), ratings of arousal were lower with the CI alone relative to both the HA alone and CI + HA conditions.

### Degree of Acoustic Hearing Loss and Device Configuration

**Figure 5.**
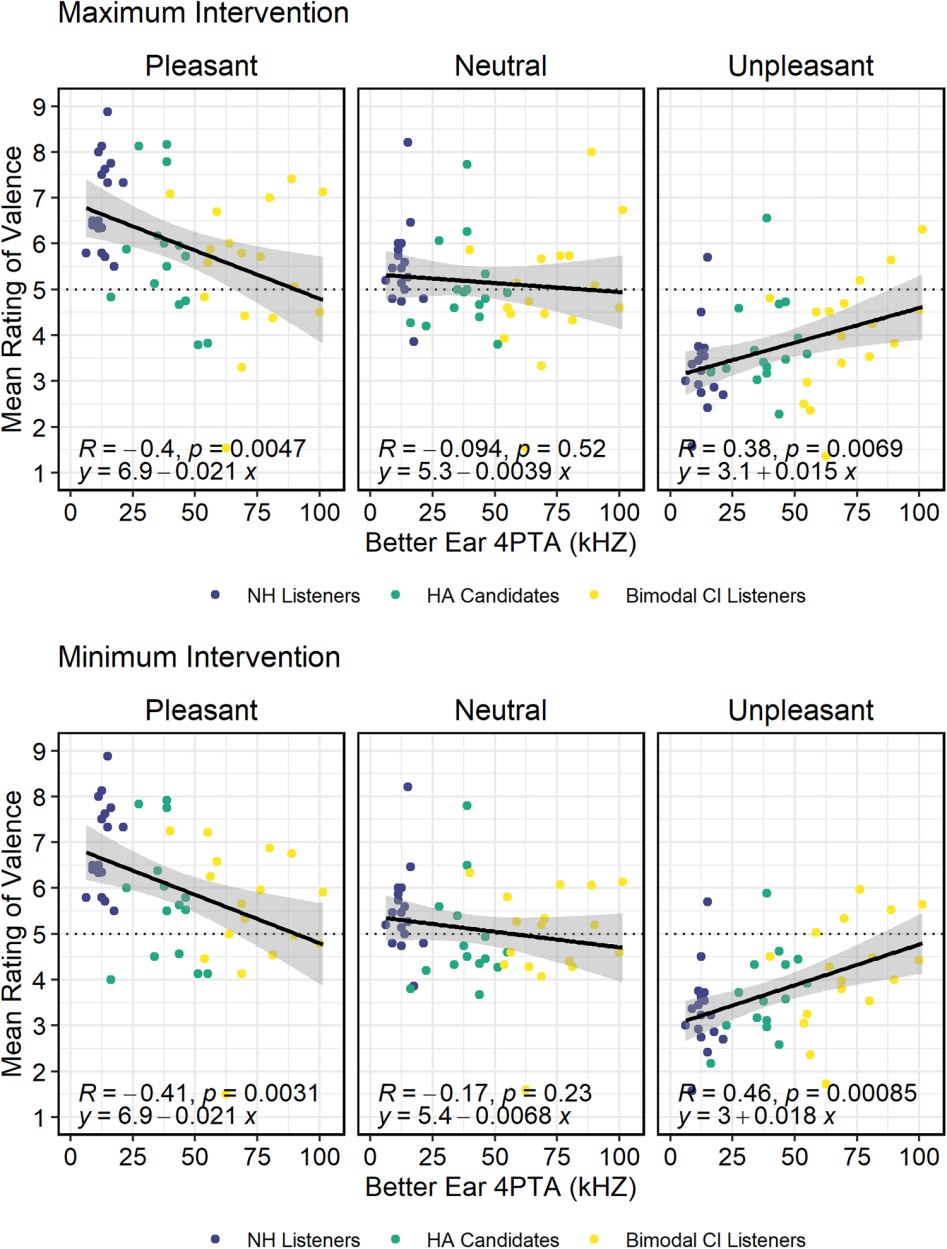
Relationship between rating of valence of pleasant, neutral, and unpleasant stimuli as a function of better ear, pure-tone average (500, 1000, 2000, and 4000 Hz) for participants using minimal intervention (unilateral HA or HA alone; top panel) or maximal intervention (bilateral HA or CI + HA; bottom panel).

To explore the relationship between degree of hearing loss on ratings of valence, exploratory correlation analyses were conducted between a participant's better ear, acoustic, pure-tone average (500, 1000, 2000, 4000 Hz) and ratings of valence, either with a minimal intervention (unilateral HA or HA alone) or with a maximum intervention (HA alone or CI + HA configuration). Mean scores in each condition/category combination were examined to preserve the data in the original scale. Data from listeners with NH was always unaided. The results, displayed in Figure 5, reveal significant negative correlations between degree of hearing loss and ratings of valence in response to pleasant sounds (*p* < 0.01) and positive correlations between degree of hearing loss and ratings of valence in response to unpleasant sounds (*p* < 0.01). Importantly, the pattern of results was the same for both device configurations (minimal/maximal). These data indicate that, regardless of device configuration, listeners with more hearing loss were more likely to rate valenced sounds as less extreme (less pleasant or less unpleasant) than listeners with better acoustic hearing thresholds.

## Discussion

The purpose of this project was two-fold: 1) to evaluate the between-group differences in emotional responses to non-speech sounds between listeners with normal hearing, hearing aid candidates, and bimodal cochlear-implant listeners and 2) to evaluate the effects of device configuration on emotional responses to non-speech sounds. Consistent with previous work (Picou, 2016; Picou & Buono, 2018; Picou, Rakita, et al., 2021), ratings of arousal were largely unaffected by hearing loss or device configuration. The limited findings related to ratings of arousal might be due to the importance of level as a cue for arousal (Buono et al., 2021; Ma et al., 2012) and listeners with hearing loss were always tested with an assistive hearing device, potentially limiting differences in loudness between groups. The following discussion focuses primarily on ratings of valence and addresses the effects of group membership and device configuration separately.

### Group Membership

The results of this study replicate those by (Picou, 2016; Picou, Rakita, et al., 2021), demonstrating that listeners with hearing loss exhibited reduced ratings of valence in response to non-speech sounds. The results also extend existing literature by demonstrating that bimodal CI listeners exhibit emotional responses that are similar to their HA-candidate peers. That is, emotional responses are also reduced in bimodal CI listeners, presumably as a result of their hearing acuity and the limited access to acoustic cues important for emotion perception.

Consistent with existing literature with HA users (Picou & Buono, 2018), these findings confirm that people who have higher unaided hearing thresholds were more likely to provide ratings of valence that were less extreme (less pleasant and less unpleasant) relative to people with better hearing thresholds. Interestingly, the relationship in the current study is nearly identical to that reported by Picou and Buono (2018). Specifically, the relationship between pure-tone average and ratings of valence of pleasant stimuli is described in the current study by the following formula: valence rating = −0.02x + 6.9, where x = pure-tone average, and by Picou and Buono (2018) as: valence rating = −0.02x + 6.97. A similar pattern emerges for ratings in response to unpleasant sounds, which is described in the current study by the formula: valence rating = 0.015x + 3.1, and by Picou and Buono (2018) as: valence rating = 0.01x + 3.22. Together, these data indicate listeners with more significant hearing loss are more likely to rate sounds as less extreme (less pleasant, less unpleasant) relative to their peers with better hearing.

Note that the relationships Picou and Buono (2018) described were all unaided, whereas the current study was aided (bilateral for HA candidates and CI + HA for bimodal CI listeners). The similarity between the relationships in the two studies suggests that hearing loss intervention does not mitigate the relationship between degree of hearing loss and ratings of valence. If an intervention designed to improve audibility (e.g., HAs) improved ratings of valence, the relationship between ratings and hearing loss would change (e.g., become non-significant or have a shallower slope). The non-significant effects of device configuration suggest that the effects of group membership might not be attributable only to differences in audibility of the cues that code for emotion (reviewed above). Indeed, existing evidence in the literature suggests there are more central changes in emotion processing for adults with hearing loss that are not fully attributable to sensory processing. For example, CI users demonstrate neurophysiological changes in late electrophysiological components relative to adults with NH (Deroche et al., 2019) and HA candidates demonstrate cortical changes in emotion perception, as measured using functional imaging technique (Husain et al., 2014).

### Device Configuration

#### Hearing Aid Candidates

A secondary purpose of this study was to evaluate the potential for changing the device configuration to affect emotional responses. The findings suggest that, for HA candidates, using unilateral or bilateral HAs did not affect emotional responses. The results demonstrate that the second HA did not contribute to ratings of valence or arousal for HA candidates. These data demonstrate that the clinical recommendation of fitting bilateral hearing aids for people with bilateral hearing loss is not contraindicated by the effect of the number of devices on emotional responses. That is, the number of hearing aids is irrelevant to the ratings of valence recorded in response to non-speech sounds. In a recent study by D'Onofrio and Gifford (2021), listeners with normal hearing likewise did not demonstrate improvement in musical sound quality ratings when listening with two ears versus one. Thus, there may simply be little added benefit for subjective judgements of sound (i.e., sound quality, emotional responses) when the signal presented across ears is of the same modality and similar quality (two normal hearing ears, D’Onofrio and Gifford, 2021; two HA ears with symmetrical hearing loss, current study). An additional explanation for the non-significant differences between responses with one and two HAs might be that bilateral benefits would only be expected for listeners with more significant hearing loss, as has been demonstrated in speech recognition tasks in the laboratory (e.g., Ricketts et al., 2019). Although a reasonable speculation, the data from the current study do not support bilateral benefits as dependent on degree of hearing loss.

#### Bimodal CI Listeners

To our knowledge, our data provide the first examination of bimodal CI listeners’ emotional responses to emotional stimuli. Our data suggest that bimodal stimulation interacts with emotional stimuli to affect the emotional response in a manner that differs from emotion in response to music. In the current study, ratings of valence were higher in configurations without a HA. That is, adding a HA to the contralateral ear of the CI participants did not improve ratings of valence; instead, adding a HA to the non-implanted ear had a negative effect on ratings of valence. These data were surprising as they are inconsistent with the large body of evidence demonstrating bimodal benefit shown for speech recognition (e.g., Dunn et al., 2005; Gifford et al., 2018; Gifford & Dorman, 2019; Potts et al., 2009; Sladen et al., 2018), perception of suprasegmental features of speech (e.g., Davidson et al., 2019; Most, Harel, et al., 2011), music perception (e.g., Cheng et al., 2018; Crew et al., 2015; Cullington & Zeng, 2011; Dorman et al., 2008; El Fata et al., 2009; Kong et al., 2005, 2012; Plant & Babic, 2016; Sucher & McDermott, 2009), emotion recognition of speech sounds (Most, Gaon-Sivan, et al., 2011), musical sound quality (D'Onofrio & Gifford, 2021) and musical emotion perception (Giannantonio et al., 2015; Shirvani et al., 2016). The findings are also inconsistent with the findings by D’Onofrio et al. (2020), who reported the addition of a contralateral hearing aid for a CI user allowed bimodal CI listeners to use both tempo and mode cues while rating valence of music, whereas ratings were based primarily on tempo when using a CI alone.

Reasons for this discrepancy are unclear and could be related to how the two modalities, acoustic and electric, might interfere with emotion. Some have suggested that integration of acoustic-electric hearing may be better when combined in the same ear as opposed to across ears for vowel recognition (Fu et al., 2017). Other studies, however, have shown no differences in integration efficiency of acoustic-electric hearing for word and sentence recognition in quiet and noise (Sheffield et al., 2015; Willis et al., 2020). The performance of bimodal listening has been previously discussed to be dependent on the effectiveness of the modalities (CI & HA) integrating with each other, and that the performances of the two should complement each other (Yoon et al., 2015).

There is also a possibility that there is conflicting middle- to high-frequency information between HAs and CI (Mok et al., 2006). For bimodal CI listeners, the CI provides both low- and high-frequency stimulation; however, amplification via the HA in the contralateral ear is often limited to low-frequency information, as these individuals typically have sloping losses. While the added amplification using the current HA fitting practices may provide important acoustic cues (e.g., F0, temporal fine structure) for the interpretation of some stimuli (e.g., speech recognition, music perception, musical emotion perception), it may actually be counterproductive for emotional responses to non-speech sounds. That is, the net effect of contralateral amplification fit to NAL-NL2 targets could simply be a boost in overall loudness (via a “doubling” of low-frequency information; acoustic + electric stimulation) that results in a relative *decrease* of important high-frequency information (via the CI). Such a perceptual decrease of high-frequency information could be at least partly responsible for the reduced emotional responses in the CI + HA condition as demonstrated here, which would be consistent with the documented negative effects on emotional responses of increasing overall stimulus presentation level (Picou, Rakita, et al., 2021) and reducing high-frequency content (Buono et al., 2021). Future research is warranted to further examine this relationship in adults using bimodal stimulation, specifically investigating whether reduced acoustic-electric overlap across ears and alternative prescriptive fitting formulae might yield improved emotional responses.

### Limitations and Future Directions

There are several limitations worthy of noting. First, the study design did not explicitly include an evaluation of the device configurations relative to unaided listening situations. Second, all participants were fit with research HAs and not all participants had prior experience with this aid or any HA. Goy et al. (2018) evaluated emotion recognition with participants’ own HAs, in part to ensure they had experience with the device settings. Unfortunately, some of the participants in that study were used to amplification that was below prescriptive targets, especially in the low-frequency region. Thus, future research is warranted to evaluate the effects of HA use on emotion perception, both recognition and valence ratings, when the HAs are matched to validated prescriptive targets and when the participants have had sufficient experience with the settings.

Third, group differences in age and gender were evident across the three groups in this study. Reported differences in gender for valence ratings of the stimuli used in this study are mixed in the extant literature for these stimuli; some investigators report no gender effects with the IADS non-speech sounds (Bradley & Lang, 2000) or music (Lundqvist et al., 2009), whereas others report females are more likely to rate unpleasant non-speech sounds with lower ratings of valence than males are (Picou & Buono, 2018). In the current study, the NH group consisted of more females than did either of the two groups of HI participants. Thus, it is possible the effects of group membership or PTA in this study might be attributable to gender differences, but only for unpleasant sounds.

The other factor of interest where groups were not matched is age; specifically, the HA candidate group was, on average, approximately 9 years older than the other groups. However, this seems unlikely to have affected our results because differences in ratings of valence related to age have been small and non-significant with these stimuli (Picou, 2016; Picou & Buono, 2018). Moreover, aging is generally associated with a positivity effect, where pleasant stimuli are rated as more pleasant by older adults than by younger ones (Backs et al., 2005; Grühn & Scheibe, 2008). In the context of the current study, the older group of participants rated pleasant stimuli as less pleasant than their peers with better hearing. Thus, it seems unlikely group differences in age contribute to the reported findings.

Fourth, the study stimuli, while all ‘non-speech sounds’ did include music samples and much of the existing work with non speech sounds are focused on music. Music and other non-speech sounds might have distinct emotional effects that warrant further investigation. For example, the results of this study are inconsistent with existing CI literature where CI users do not demonstrate ratings of valence relative to listeners with NH in response to music (Ambert-Dahan et al., 2015). Although exploratory analyses (not reported here) of the current data set revealed the same pattern of results for the music and non-music sounds in the study, it seems difficult to draw conclusions about the distinction in ratings of valence between music and non-music because the current data set included only 8 music sounds out of 75 total sounds. Thus future work is warranted to disentangle emotional responses to music from other non-speech sounds.

## Conclusions

Even with assistive hearing devices (HAs and CIs), adults with hearing loss demonstrate a reduced range of valence ratings in response to non-speech sounds, as evidenced by less extreme ratings (less pleasant and less unpleasant) than their similarly aged peers with normal hearing. Those with more significant acoustic hearing loss were more likely to exhibit less extreme ratings of valence than were those with better unaided acoustic thresholds. This finding has important implications for the psychosocial well-being of adults with hearing loss, where emotional responses to sounds have been linked to isolation and loneliness (Picou & Buono, 2018), stress recovery (Sandstrom & Russo, 2010), in addition to focused attention and enhanced memory (e.g., Kensinger, 2009). Thus, rehabilitation for adults with hearing loss should consider emotion perception. Based on the cues that support ratings of valence, it is likely that interventions that provide auditory access to broadband stimuli would improve ratings of valence in response to non-speech sounds. Yet, the results of this study do not provide insight into optimizing device configuration; ratings of valence were similar with one and two HAs for hearing aid candidates. For bimodal CI listeners, the conditions with the HA resulted in overall lower ratings of valence and arousal. These data would suggest CI + HA listening may not be optimal for emotional responses to non-speech sounds, despite clear advantages in other auditory domains. Combined, these data support the need for further investigation into hearing device optimization to improve ratings of valence in response to non-speech sounds for adults with sensorineural hearing loss.

## Supplementary Material

Supplementary material

## References

[bibr1-23312165221083091] Alcántara J. Moore B. Kühnel V. Launer S. (2003). Evaluation of the noise reduction system in a commercial digital hearing aid. International Journal of Audiology, 42(1), 34–42. 10.3109/14992020309056083 12564514

[bibr2-23312165221083091] Alvarsson J. J. Wiens S. Nilsson M. E. (2010). Stress recovery during exposure to nature sound and environmental noise. International Journal of Environmental Research and Public Health, 7(3), 1036–1046. 10.3390/ijerph7031036 20617017 PMC2872309

[bibr3-23312165221083091] Ambert-Dahan E. Giraud A.-L. Sterkers O. Samson S. (2015). Judgment of musical emotions after cochlear implantation in adults with progressive deafness. Frontiers in Psychology, 6, 181. 10.3389/fpsyg.2015.00181 25814961 PMC4357245

[bibr4-23312165221083091] Atias D. Todorov A. Liraz S. Eidinger A. Dror I. Maymon Y. Aviezer H. (2019). Loud and unclear: Intense real-life vocalizations during affective situations are perceptually ambiguous and contextually malleable. Journal of Experimental Psychology: General, 148(10), 1842–1848. 10.1037/xge0000535 30589289

[bibr5-23312165221083091] Backs R. W. da Silva S. P. Han K. (2005). A comparison of younger and older adults’ self-assessment manikin ratings of affective pictures. Experimental Aging Research, 31(4), 421–440. 10.1080/03610730500206808 16147461

[bibr6-23312165221083091] Banse R. Scherer K. R. (1996). Acoustic profiles in vocal emotion expression. Journal of Personality and Social Psychology, 70(3), 614–636. 10.1037/0022-3514.70.3.614 8851745

[bibr7-23312165221083091] Bates D. Maechler M. Bolker B. Walker S. (2015). Fitting linear mixed-effects models using {lme4}. Journal of Statistical Software, 67(1), 1–48. 10.18637/jss.v067.i01

[bibr8-23312165221083091] Baumeister R. F. Bratslavsky E. Finkenauer C. Vohs K. D. (2001). Bad is stronger than good. Review of General Psychology, 5(4), 323–370. 10.1037//1089-2680.5.4.323

[bibr9-23312165221083091] Benjamini Y. Hochberg Y. (1995). Controlling the false discovery rate: A practical and powerful approach to multiple testing. Journal of the Royal Statistical Society, 57(1), 289–300. 10.1111/j.2517-6161.1995.tb02031.x

[bibr10-23312165221083091] Boymans M. Goverts S. T. Kramer S. E. Festen J. M. Dreschler W. A. (2008). A prospective multi-centre study of the benefits of bilateral hearing aids. Ear and Hearing, 29(6), 930–941. 10.1097/AUD.0b013e31818713a8 18998242

[bibr11-23312165221083091] Bradley M. M. Codispoti M. Cuthbert B. N. Lang P. J. (2001). Emotion and motivation I: Defensive and appetitive reactions in picture processing. Emotion (Washington, D.C.), 1(3), 276–298. 10.1037//1528-3542.1.3.276 12934687

[bibr12-23312165221083091] Bradley M. M. Lang P. J. (1994). Measuring emotion: The self-assessment manikin and the semantic differential. Journal of Behavior Therapy and Experimental Psychiatry, 25(1), 49–59. 10.1016/0005-7916(94)90063-9 7962581

[bibr13-23312165221083091] Bradley M. M. Lang P. J. (2000). Affective reactions to acoustic stimuli. Psychophysiology, 37(02), 204–215. 10.1111/1469-8986.3720204 10731770

[bibr14-23312165221083091] Bradley M. M. Lang P. J. (2007). The International Affective Digitized Sounds (IADS-2): Affective ratings of sounds and instruction manual. *University of Florida, Gainesville, FL, Tech. Rep. B-3*.

[bibr15-23312165221083091] Buono G. H. Crukley J. Hornsby B. W. Picou E. M. (2021). Loss of high-or low-frequency audibility can partially explain effects of hearing loss on emotional responses to non-speech sounds. Hearing Research, 401, 108153. 10.1016/j.heares.2020.108153 33360158

[bibr16-23312165221083091] Caldwell M. Rankin S. K. Jiradejvong P. Carver C. Limb C. J. (2015). Cochlear implant users rely on tempo rather than on pitch information during perception of musical emotion. Cochlear Implants International, 16(sup3), S114–S120. 10.1179/1467010015Z.000000000265 26561882

[bibr17-23312165221083091] Chatterjee M. Peng S.-C. (2008). Processing F0 with cochlear implants: Modulation frequency discrimination and speech intonation recognition. Hearing Research, 235(1-2), 143–156. 10.1016/j.heares.2007.11.004 18093766 PMC2237883

[bibr18-23312165221083091] Chatterjee M. Zion D. J. Deroche M. L. Burianek B. A. Limb C. J. Goren A. P. Kulkarni A. M. Christensen J. A. (2015). Voice emotion recognition by cochlear-implanted children and their normally-hearing peers. Hearing Research, 322, 151–162. 10.1016/j.heares.2014.10.003 25448167 PMC4615700

[bibr19-23312165221083091] Cheng X. Liu Y. Wang B. Yuan Y. Galvin J. J. Fu Q.-J. Shu Y. Chen B. (2018). The benefits of residual hair cell function for speech and music perception in pediatric bimodal cochlear implant listeners. Neural Plasticity, 2018, 4610592. 10.1155/2018/4610592 29849556 PMC5925034

[bibr20-23312165221083091] Christensen J. A. Sis J. Kulkarni A. M. Chatterjee M. (2019). Effects of age and hearing loss on the recognition of emotions in speech. Ear and Hearing, 40(5), 1069–1083. 10.1097/AUD.0000000000000694 30614835 PMC6606405

[bibr21-23312165221083091] Crew J. D. Galvin J. J. III Landsberger D. M. Fu Q.-J. (2015). Contributions of electric and acoustic hearing to bimodal speech and music perception. PLoS One, 10(3), e0120279. 10.1371/journal.pone.0120279. 25790349 PMC4366155

[bibr22-23312165221083091] Cullington H. E. Zeng F.-G. (2011). Comparison of bimodal and bilateral cochlear implant users on speech recognition with competing talker, music perception, affective prosody discrimination and talker identification. Ear and Hearing, 32(1), 16–30. 10.1097/AUD.0b013e3181edfbd2 21178567 PMC3059251

[bibr23-23312165221083091] Dalton D. Cruickshanks K. Klein B. Klein R. Wiley T. Nondahl D. (2003). The impact of hearing loss on quality of life in older adults. The Gerontologist, 43(5), 661–668. 10.1093/geront/43.5.661 14570962

[bibr24-23312165221083091] Damm S. A. Sis J. L. Kulkarni A. M. Chatterjee M. (2019). How vocal emotions produced by children with cochlear implants are perceived by their hearing peers. Journal of Speech, Language, and Hearing Research, 62(10), 3728–3740. 10.1044/2019_JSLHR-S-18-0497 PMC7201339 31589545

[bibr25-23312165221083091] Davidson L. S. Geers A. E. Uchanski R. M. Firszt J. B. (2019). Effects of early acoustic hearing on speech perception and language for pediatric cochlear implant recipients. Journal of Speech, Language, and Hearing Research, 62(9), 3620–3637. 10.1044/2019_JSLHR-H-18-0255 PMC6808345 31518517

[bibr26-23312165221083091] Deroche M. L. Felezeu M. Paquette S. Zeitouni A. Lehmann A. (2019). Neurophysiological differences in emotional processing by cochlear implant users, extending beyond the realm of speech. Ear and Hearing, 40(5), 1197–1209. 10.1097/AUD.0000000000000701 30762600

[bibr27-23312165221083091] D’Onofrio K. L. Caldwell M. Limb C. Smith S. Kessler D. M. Gifford R. H. (2020). Musical emotion perception in bimodal patients: Relative weighting of musical mode and tempo cues. Frontiers in Neuroscience, 14, 114. 10.3389/fnins.2020.00114 32174809 PMC7054459

[bibr28-23312165221083091] D'Onofrio K. L. Gifford R. H. (2021). Bimodal benefit for music perception: Effect of acoustic bandwidth. Journal of Speech, Language, and Hearing Research, 64(4), 1341–1353. 10.1044/2020_JSLHR-20-00390 PMC8608177 33784471

[bibr29-23312165221083091] Dorman M. F. Gifford R. H. (2010). Combining acoustic and electric stimulation in the service of speech recognition. International Journal of Audiology, 49(12), 912–919. 10.3109/14992027.2010.509113 20874053 PMC2992956

[bibr30-23312165221083091] Dorman M. F. Gifford R. H. Spahr A. J. McKarns S. A. (2008). The benefits of combining acoustic and electric stimulation for the recognition of speech, voice and melodies. Audiology and Neurotology, 13(2), 105–112. 10.1159/000111782 18057874 PMC3559130

[bibr31-23312165221083091] Dunn C. C. Tyler R. S. Witt S. A. (2005). Benefit of wearing a hearing aid on the unimplanted ear in adult users of a cochlear implant. Journal of Speech, Language, and Hearing Research, 48, 668–680. 10.1044/1092-4388(2005/046) 16197280

[bibr32-23312165221083091] Dupuis K. Pichora-Fuller M. K. (2014). Intelligibility of emotional speech in younger and older adults. Ear and Hearing, 35(6), 695–707. 10.1097/AUD.0000000000000082 25127327

[bibr33-23312165221083091] Eerola T. Vuoskoski J. K. (2013). A review of music and emotion studies: Approaches, emotion models, and stimuli. Music Perception: An Interdisciplinary Journal, 30(3), 307–340. 10.1525/MP.2012.30.3.307

[bibr34-23312165221083091] El Fata F. James C. J. Laborde M.-L. Fraysse B. (2009). How much residual hearing is ‘useful’for music perception with cochlear implants? Audiology and Neurotology, 14(Suppl. 1), 14–21. 10.1159/000206491 19390171

[bibr35-23312165221083091] Erdman S. A. Sedge R. K. (1981). Subjective comparisons of binaural versus monaural amplification. Ear and Hearing, 2(5), 225–229. 10.1097/00003446-198109000-00009 7297789

[bibr36-23312165221083091] Faith M. Thayer J. F. (2001). A dynamical systems interpretation of a dimensional model of emotion. Scandinavian Journal of Psychology, 42(2), 121–133. 10.1111/1467-9450.00221 11321635

[bibr37-23312165221083091] Fredrickson B. L. (2001). The role of positive emotions in positive psychology: The broaden-and-build theory of positive emotions. American Psychologist, 56(3), 218–226. 10.1O37//0OO3-O66X.56.3.218 PMC3122271 11315248

[bibr38-23312165221083091] Fredrickson B. L. Branigan C. (2005). Positive emotions broaden the scope of attention and thought–action repertoires. Cognition & Emotion, 19(3), 313–332. 10.1080/02699930441000238 21852891 PMC3156609

[bibr39-23312165221083091] Freyaldenhoven M. C. Plyler P. N. Thelin J. W. Burchfield S. B. (2006). Acceptance of noise with monaural and binaural amplification. Journal of the American Academy of Audiology, 17(9), 659–666. 10.3766/jaaa.17.9.5 17039768

[bibr40-23312165221083091] Fu Q.-J. Galvin J. J. Wang X. (2017). Integration of acoustic and electric hearing is better in the same ear than across ears. Scientific Reports, 7(1), 1–9. 10.1038/s41598-017-12298-3 28970567 PMC5624923

[bibr41-23312165221083091] Giannantonio S. Polonenko M. J. Papsin B. C. Paludetti G. Gordon K. A. (2015). Experience changes how emotion in music is judged: Evidence from children listening with bilateral cochlear implants, bimodal devices, and normal hearing. PLoS One, 10(8), e0136685. 10.1371/journal.pone.0136685 26317976 PMC4552689

[bibr42-23312165221083091] Gifford R. H. Dorman M. F. (2019). Bimodal hearing or bilateral cochlear implants? Ask the patient. Ear and Hearing, 40(3), 501–517. 10.1097/AUD.0000000000000657 30285977 PMC6447482

[bibr43-23312165221083091] Gifford R. H. Noble J. H. Camarata S. M. Sunderhaus L. W. Dwyer R. T. Dawant B. M. Dietrich M. S. Labadie R. F. (2018). The relationship between spectral modulation detection and speech recognition: Adult versus pediatric cochlear implant recipients. Trends in Hearing, 22, 1–14. 10.1177/2331216518771176 PMC5949922 29716437

[bibr44-23312165221083091] Gifford R. H. Sunderhaus L. Sheffield S. (2021). Bimodal hearing with pediatric cochlear implant recipients: Effect of acoustic bandwidth. Otology & Neurotology, 42(10), S19–S25. 10.1097/MAO.0000000000003375 34766940

[bibr45-23312165221083091] Gosselin N. Peretz I. Noulhiane M. Hasboun D. Beckett C. Baulac M. Samson S. (2005). Impaired recognition of scary music following unilateral temporal lobe excision. Brain, 128(3), 628–640. 10.1093/brain/awh420 15699060

[bibr46-23312165221083091] Goudbeek M. Scherer K. (2010). Beyond arousal: Valence and potency/control cues in the vocal expression of emotion. The Journal of the Acoustical Society of America, 128(3), 1322–1336. 10.1121/1.3466853 20815467

[bibr47-23312165221083091] Goy H. Pichora-Fuller K. M. Singh G. Russo F. A. (2018). Hearing aids benefit recognition of words in emotional speech but not emotion identification. Trends in Hearing, 22, 1–16. 10.1177/2331216518801736 PMC6156210 30249171

[bibr48-23312165221083091] Grühn D. Scheibe S. (2008). Age-related differences in valence and arousal ratings of pictures from the International Affective Picture System (IAPS): Do ratings become more extreme with age? Behavior Research Methods, 40(2), 512–521. https://doi.org/10.3758/BRM.40.2.512 18522062

[bibr49-23312165221083091] Hawkins D. B. Yacullo W. S. (1984). Signal-to-noise ratio advantage of binaural hearing aids and directional microphones under different levels of reverberation. Journal of Speech and Hearing Disorders, 49(3), 278–286. 10.1044/jshd.4903.278 6748623

[bibr50-23312165221083091] Hawthorne G. (2008). Perceived social isolation in a community sample: Its prevalence and correlates with aspects of peoples’ lives. Social Psychiatry and Psychiatric Epidemiology, 43(2), 140–150. 10.1007/s00127-007-0279-8 17994175

[bibr51-23312165221083091] Holder J. T. Reynolds S. M. Sunderhaus L. W. Gifford R. H. (2018). Current profile of adults presenting for preoperative cochlear implant evaluation. Trends in Hearing, 22, 1–16. 10.1177/2331216518755288 PMC6027468 29441835

[bibr52-23312165221083091] Hsiao F. Gfeller K. (2012). Music perception of cochlear implant recipients with implications for music instruction: A review of the literature. Update: Applications of Research in Music Education, 30(2), 5–10. 10.1177/8755123312437050 23469365 PMC3587135

[bibr53-23312165221083091] Humes L. E. Roberts L. (1990). Speech-recognition difficulties of the hearing-impaired elderly: The contributions of audibility. Journal of Speech, Language and Hearing Research, 33(4), 726–735. 10.1044/jshr.3304.726 2273886

[bibr54-23312165221083091] Humes L. E. Wilson D. L. Barlow N. N. Garner C. (2002). Changes in hearing-aid benefit following 1 or 2 years of hearing-aid use by older adults. Journal of Speech, Language and Hearing Research, 45(4), 772–782. 10.1044/1092-4388(2002/062) 12199406

[bibr55-23312165221083091] Husain F. T. Carpenter-Thompson J. R. Schmidt S. A. (2014). The effect of mild-to-moderate hearing loss on auditory and emotion processing networks. Frontiers in Systems Neuroscience, 8, 1–13. 10.3389/fnsys.2014.00010 24550791 PMC3912518

[bibr56-23312165221083091] Ilie G. Thompson W. F. (2006). A comparison of acoustic cues in music and speech for three dimensions of affect. Music Perception: An Interdisciplinary Journal, 23(4), 319–330. 10.1525/mp.2006.23.4.319

[bibr57-23312165221083091] Jiam N. Caldwell M. Deroche M. Chatterjee M. Limb C. (2017). Voice emotion perception and production in cochlear implant users. Hearing Research, 352, 30–39. 10.1016/j.heares.2017.01.006 PMC5937709 28088500

[bibr58-23312165221083091] Juslin P. N. Laukka P. (2003). Communication of emotions in vocal expression and music performance: Different channels, same code? Psychological Bulletin, 129(5), 770. 10.1037/0033-2909.129.5.770 12956543

[bibr59-23312165221083091] Keidser G. Dillon H. Carter L. O’Brien A. (2012). NAL-NL2 empirical adjustments. Trends in Amplification, 16(4), 211–223. 10.1177/1084713812468511 23203416 PMC4040825

[bibr60-23312165221083091] Kensinger E. A. (2009). Remembering the details: Effects of emotion. Emotion Review, 1(2), 99–113. 10.1177/1754073908100432 19421427 PMC2676782

[bibr61-23312165221083091] Kiss I. Ennis T. (2001). Age-related decline in perception of prosodic affect. Applied Neuropsychology, 8(4), 251–254. 10.1207/S15324826AN0804_9 11989730

[bibr62-23312165221083091] Köbler S. Rosenhall U. Hansson H. (2001). Bilateral hearing aids-effects and consequences from a user perspective. Scandinavian Audiology, 30(4), 223–235. 10.1080/01050390152704742 11845991

[bibr63-23312165221083091] Kong Y.-Y. Carlyon R. P. (2007). Improved speech recognition in noise in simulated binaurally combined acoustic and electric stimulation. The Journal of the Acoustical Society of America, 121(6), 3717–3727. 10.1121/1.2717408 17552722

[bibr64-23312165221083091] Kong Y.-Y. Cruz R. Jones J. A. Zeng F.-G. (2004). Music perception with temporal cues in acoustic and electric hearing. Ear and Hearing, 25(2), 173–185. https://doi.org/10.1097/01.AUD.0000120365.97792.2F 15064662

[bibr65-23312165221083091] Kong Y.-Y. Mullangi A. Marozeau J. (2012). Timbre and speech perception in bimodal and bilateral cochlear-implant listeners. Ear and Hearing, 33(5), 645–659. 10.1097/AUD.0b013e318252caae 22677814 PMC3428469

[bibr66-23312165221083091] Kong Y.-Y. Stickney G. S. Zeng F.-G. (2005). Speech and melody recognition in binaurally combined acoustic and electric hearing. The Journal of the Acoustical Society of America, 117(3), 1351–1361. 10.1121/1.1857526 15807023

[bibr67-23312165221083091] Kramer S. E. Kapteyn T. S. Kuik D. J. Deeg D. J. (2002). The association of hearing impairment and chronic diseases with psychosocial health status in older age. Journal of Aging and Health, 14(1), 122–137. 10.1177/089826430201400107 11892756

[bibr68-23312165221083091] Laukka P. Juslin P. Bresin R. (2005). A dimensional approach to vocal expression of emotion. Cognition & Emotion, 19(5), 633–653. 10.1080/02699930441000445

[bibr69-23312165221083091] Lenth R. (2019). emmeans: Estimated Marginal Means, aka Least-Squares Means. R package version 1.4. https://CRAN.R-project.org/package=emmeans

[bibr70-23312165221083091] Lundqvist L.-O. Carlsson F. Hilmersson P. Juslin P. (2009). Emotional responses to music: Experience, expression, and physiology. Psychology of Music, 37(1), 61–90. 10.1177/0305735607086048

[bibr71-23312165221083091] Luo X. Fu Q.-J. Galvin J. J. (2007). Vocal emotion recognition by normal-hearing listeners and cochlear implant users. Trends in Amplification, 11(4), 301–315. 10.1177/1084713807305301 18003871 PMC4111530

[bibr72-23312165221083091] Ma C. Shaw G. M. Scheuerle A. E. Canfield M. A. Carmichael S. L. (2012). Association of microtia with maternal nutrition. Birth Defects Research Part A: Clinical and Molecular Teratology, 94(12), 1026–1032. 10.1002/bdra.23053 22821770

[bibr73-23312165221083091] Ma W. Thompson W. F. (2015). Human emotions track changes in the acoustic environment. Proceedings of the National Academy of Sciences, 112(47), 14563–14568. 10.1073/pnas.1515087112/-/DCSupplemental PMC4664334 26553987

[bibr74-23312165221083091] Mok M. Grayden D. Dowell R. C. Lawrence D. (2006). Speech perception for adults who use hearing aids in conjunction with cochlear implants in opposite ears. Journal of Speech, Language & Hearing Research, 49(2), 338–351. 10.1044/1092-4388(2006/027) 16671848

[bibr75-23312165221083091] Most T. Aviner C. (2009). Auditory, visual, and auditory–visual perception of emotions by individuals with cochlear implants, hearing aids, and normal hearing. Journal of Deaf Studies and Deaf Education, 14(4), 449–464. 10.1093/deafed/enp007 19398533

[bibr76-23312165221083091] Most T. Gaon-Sivan G. Shpak T. Luntz M. (2011). Contribution of a contralateral hearing aid to perception of consonant voicing, intonation, and emotional state in adult cochlear implantees. Journal of Deaf Studies and Deaf Education, 17(2), 244–258. 10.1093/deafed/enr046 22057984

[bibr77-23312165221083091] Most T. Harel T. Shpak T. Luntz M. (2011). Perception of suprasegmental speech features via bimodal stimulation: Cochlear implant on one ear and hearing aid on the other. Journal of Speech, Language & Hearing Research, 54(2), 668–678. 10.1044/1092-4388(2010/10-0071) 20844254

[bibr78-23312165221083091] Osgood C. E. Suci G. J. Tannenbaum P. H. (1957). The measurement of meaning (Vol. 47). University of Illinois Press.

[bibr79-23312165221083091] Paquette S. Ahmed G. Goffi-Gomez M. Hoshino A. Peretz I. Lehmann A. (2018). Musical and vocal emotion perception for cochlear implants users. Hearing Research, 370, 272–282. 10.1016/j.heares.2018.08.009 30181063

[bibr80-23312165221083091] Paulmann S. Pell M. D. Kotz S. A. (2008). How aging affects the recognition of emotional speech. Brain and Language, 104(3), 262–269. 10.1016/j.bandl.2007.03.002 17428529

[bibr81-23312165221083091] Pell M. D. Paulmann S. Dara C. Alasseri A. Kotz S. A. (2009). Factors in the recognition of vocally expressed emotions: A comparison of four languages. Journal of Phonetics, 37(4), 417–435. 10.1016/j.wocn.2009.07.005

[bibr82-23312165221083091] Peretz I. Gagnon L. Bouchard B. (1998). Music and emotion: Perceptual determinants, immediacy, and isolation after brain damage. Cognition, 68(2), 111–141. 10.1016/S0010-0277(98)00043-2 9818509

[bibr83-23312165221083091] Peters R. W. Moore B. C. J. Baer T. (1998). Speech reception thresholds in noise with and without spectral and temporal dips for hearing-impaired and normally hearing people. The Journal of the Acoustical Society of America, 103, 577–587. 10.1121/1.421128 9440343

[bibr84-23312165221083091] Picou E. M. (2016). How hearing loss and age affect emotional responses to nonspeech sounds. Journal of Speech, Language, and Hearing Research, 59(5), 1233–1246. 10.1044/2016_JSLHR-H-15-0231 27768178

[bibr85-23312165221083091] Picou E. M. Buono G. H. (2018). Emotional responses to pleasant sounds are related to social disconnectedness and loneliness independent of hearing loss. Trends in Hearing, 22, 1–15. 10.1177/2331216518813243 PMC6277757 30482108

[bibr86-23312165221083091] Picou E. M. Rakita L. Buono G. H. Moore T. M. (2021). Effects of increasing the overall level or fitting hearing aids on emotional responses to sounds. Trends in Hearing, 25, 1–13. 10.1177/23312165211049938 PMC8825634 34866509

[bibr87-23312165221083091] Picou E. M. Ricketts T. A. Hornsby B. W. (2013). How hearing aids, background noise, and visual cues influence objective listening effort. Ear and Hearing, 34, e52–e64. 10.1097/AUD.0b013e31827f0431 23416751

[bibr88-23312165221083091] Picou E. M. Roberts R. A. Angley G. Ricketts T. A. (2021). Applying the hearing aid fitting standard to selection for adults. *Seminars in Hearing, in review*. 10.1055/s-0042-1748874 PMC9325089 35903077

[bibr89-23312165221083091] Plant K. Babic L. (2016). Utility of bilateral acoustic hearing in combination with electrical stimulation provided by the cochlear implant. International Journal of Audiology, 55(sup2), S31–S38. 10.3109/14992027.2016.1150609 26987051

[bibr90-23312165221083091] Plomp R. (1976). Binaural and monaural speech intelligibility of connected discourse in reverberation as a function of azimuth of a single competing sound source (speech or noise). Acustica, 34, 200–211.

[bibr91-23312165221083091] Potts L. G. Skinner M. W. Litovsky R. A. Strube M. J. Kuk F. (2009). Recognition and localization of speech by adult cochlear implant recipients wearing a digital hearing aid in the nonimplanted ear (bimodal hearing). Journal of the American Academy of Audiology, 20(6), 353–373. 10.3766/jaaa.20.6.4 19594084 PMC2876351

[bibr92-23312165221083091] R Core Team (2021). *R: A language and environment for statistical computing*. Vienna, Austria. https://www.R-project.org/

[bibr93-23312165221083091] Ricketts T. A. Picou E. M. Shehorn J. Dittberner A. B. (2019). Degree of hearing loss affects bilateral hearing aid benefits in ecologically relevant laboratory conditions. Journal of Speech, Language, and Hearing Research, 62(10), 3834–3850. 10.1044/2019_JSLHR-H-19-0013 PMC7201333 31596645

[bibr94-23312165221083091] Rigo T. G. Lieberman D. A. (1989). Nonverbal sensitivity of normal-hearing and hearing-impaired older adults. Ear and Hearing, 10(3), 184–189. 10.1097/00003446-198906000-00008 2744255

[bibr95-23312165221083091] Rosslau K. Spreckelmeyer K. N. Saalfeld H. Westhofen M. (2012). Emotional and analytic music perception in cochlear implant users after optimizing the speech processor. Acta Oto-Laryngologica, 132(1), 64–71. 10.3109/00016489.2011.619569 22026456

[bibr96-23312165221083091] Sandstrom G. M. Russo F. A. (2010). Music hath charms: The effects of valence and arousal on recovery following an acute stressor. Music and Medicine, 2(3), 137–143. 10.1177/1943862110371486

[bibr97-23312165221083091] Schmidt J. Herzog D. Scharenborg O. Janse E. (2016). Do hearing aids improve affect perception? In van Dijk P. Başkent D. Gaudrain E. de Kleine E. Wagner A. Lanting C. (Eds.), Physiology, psychoacoustics and cognition in normal and impaired hearing: Advances in experimental medicine and biology (Vol. 894, pp. 47–55). Springer. 10.1007/978-3-319-25474-6_6 27080645

[bibr98-23312165221083091] Schmidt J. Janse E. Scharenborg O. (2016). Perception of emotion in conversational speech by younger and older listeners. Frontiers in Psychology, 7, 1–11. 10.3389/fpsyg.2016.00781 27303340 PMC4885861

[bibr99-23312165221083091] Schreurs K. K. Olsen W. O. (1985). Comparison of monaural and binaural hearing aid use on a trial period basis. Ear and Hearing, 6(4), 198–202. 10.1097/00003446-198507000-00005 4043573

[bibr100-23312165221083091] Sheffield S. W. Gifford R. H. (2014). The benefits of bimodal hearing: Effect of frequency region and acoustic bandwidth. Audiology and Neurotology, 19(3), 151–163. 10.1159/000357588 24556850 PMC4104148

[bibr101-23312165221083091] Sheffield S. W. Jahn K. Gifford R. H. (2015). Preserved acoustic hearing in cochlear implantation improves speech perception. Journal of the American Academy of Audiology, 26(02), 145–154. 10.3766/jaaa.26.2.5 25690775 PMC4446708

[bibr102-23312165221083091] Shirvani S. Jafari Z. Motasaddi Zarandi M. Jalaie S. Mohagheghi H. Tale M. R. (2016). Emotional perception of music in children with bimodal fitting and unilateral cochlear implant. Annals of Otology, Rhinology & Laryngology, 125(6), 470–477. 10.1177/0003489415619943 26681623

[bibr103-23312165221083091] Shirvani S. Jafari Z. Sheibanizadeh A. Zarandy M. M. Jalaie S. (2014). Emotional perception of music in children with unilateral cochlear implants. Iranian Journal of Otorhinolaryngology, 26(77), 225. 25320700 PMC4196446

[bibr104-23312165221083091] Singh G. Liskovoi A. Launer S. Russo F. A. (2019). The Emotional Communication in Hearing Questionnaire (EMO-CHeQ): Development and evaluation. Ear and Hearing, 40(2), 260–272. 10.1097/AUD.0000000000000611 29894380 PMC6400448

[bibr105-23312165221083091] Sladen D. P. Carlson M. L. Dowling B. P. Olund A. P. DeJong M. D. Breneman A. Hollander S. Beatty C. W. Neff B. A. Driscoll C. L. (2018). Cochlear implantation in adults with asymmetric hearing loss: Speech recognition in quiet and in noise, and health related quality of life. Otology & Neurotology, 39(5), 576–581. 10.1097/MAO.0000000000001763 29683995

[bibr106-23312165221083091] Sucher C. M. McDermott H. J. (2009). Bimodal stimulation: Benefits for music perception and sound quality. Cochlear Implants International, 10(S1), 96–99. 10.1002/cii.398 19230032

[bibr107-23312165221083091] Taylor S. E. (1991). Asymmetrical effects of positive and negative events: The mobilization-minimization hypothesis. Psychological Bulletin, 110(1), 67–85. 10.1037/0033-2909.110.1.67 1891519

[bibr108-23312165221083091] Vaughan-Jones R. H. Padgham N. D. Christmas H. E. Irwin J. Doig M. (1993). One aid or two?—more visits please!. The Journal of Laryngology & Otology, 107(04), 329–332. 10.1017/S0022215100122947 8320520

[bibr109-23312165221083091] Weninger F. Eyben F. Schuller B. W. Mortillaro M. Scherer K. R. (2013). On the acoustics of emotion in audio: What speech, music, and sound have in common. Frontiers in Psychology, 4, 292. 10.3389/fpsyg.2013.00292 23750144 PMC3664314

[bibr110-23312165221083091] Willis S. Moore B. C. Galvin J. J. III Fu Q.-J. (2020). Effects of noise on integration of acoustic and electric hearing within and across ears. PLoS One, 15(10), e0240752. 10.1371/journal.pone.0240752 33057396 PMC7561114

[bibr111-23312165221083091] Yoon Y.-S. Shin Y.-R. Gho J.-S. Fu Q.-J. (2015). Bimodal benefit depends on the performance difference between a cochlear implant and a hearing aid. Cochlear Implants International, 16(3), 159–167. 10.1179/1754762814Y.0000000101 25329752 PMC5847325

[bibr112-23312165221083091] Zajonc R. B. (1980). Feeling and thinking: Preferences need no inferences. American Psychologist, 35(2), 151–175. 10.1037/0003-066X.35.2.151

[bibr113-23312165221083091] Zigmond A. S. Snaith R. P. (1983). The hospital anxiety and depression scale. Acta Psychiatrica Scandinavica, 67(6), 361–370. 10.1111/j.1600-0447.1983.tb09716.x 6880820

