# Supplementary material for: Emotional Responses to Non-Speech Sounds for Hearing-aid and Bimodal Cochlear-Implant Listeners
Source: Trends Hear. 2022 Apr 18;26:23312165221083091. doi: 10.1177/23312165221083091 (PMC9019384; doi:10.1177/23312165221083091)
Supplement: Supplementary material [file appendices.docx]

| ID | Age | gender | HADS-Anxiety | HADS-Depression | Better ear PTA |
| --- | --- | --- | --- | --- | --- |
| A | 26 | F | 7 | 2 | 12.5 |
| B | 51 | F | 3 | 1 | 11.25 |
| C | 57 | F | 7 | 3 | 21.25 |
| D | 52 | F | 7 | 6 | 11.25 |
| E | 55 | F | 10 | 3 | 17.5 |
| F | 52 | M | 3 | 0 | 13.75 |
| G | 53 | F | 3 | 2 | 12.5 |
| H | 54 | F | 6 | 2 | 11.25 |
| I | 57 | F | 4 | 1 | 16.25 |
| J | 51 | F | 5 | 2 | 15 |
| K | 60 | F | 10 | 2 | 8.75 |
| L | 54 | F | 1 | 0 | 13.75 |
| M | 60 | F | 10 | 1 | 12.5 |
| N | 58 | F | 7 | 1 | 8.75 |
| O | 63 | M | 11 | 3 | 12.5 |
| P | 56 | F | 5 | 1 | 6.25 |
| Q | 79 | M | unknown | unknown | 15 |

Appendix A. Demographics and identification for listeners with normal hearing. Note: PTA = average of pure-tone thresholds at 500, 1000, 2000, and 4000 Hz, HADS = Hospital Anxiety and Depression Scale (Zigmond & Snaith, 1983)

Appendix B. Demographics and identification for hearing aid candidates. Note: PTA = average of pure-tone thresholds at 500, 1000, 2000, and 4000 Hz. HADS = Hospital Anxiety and Depression Scale (Zigmond & Snaith, 1983); HL = hearing loss; HA = hearing aid; RMS = root mean square

| ID | Age | Gender | HADS-Anxiety | HADS-Depression | Better Ear PTA | | HL Duration (years) | HA use duration (years) | Better ear RMS HA deviation from target  (.5, 1, 2, and 4 kHz) |
| --- | --- | --- | --- | --- | --- | --- | --- | --- | --- |
| AA | 65 | M | 0 | 0 | | 37.5 | 4 | 2 | 2.5 |
| BB | 62 | F | 3 | 0 | | 38.75 | 4 | None | 1.6 |
| CC | 68 | M | 1 | 0 | | 16.25 | 6 | None | 3.0 |
| DD | 62 | M | 2 | 1 | | 22.5 | 15 | 2.5 | 1.2 |
| EE | 74 | M | 3 | 3 | | 38.75 | 5 | 2 | 1.1 |
| FF | 62 | F | 6 | 2 | | 38.75 | 30 | None | 2.1 |
| GG | 60 | M | 12 | 7 | | 46.25 | 5 | 5 | 1.6 |
| HH | 55 | M | 3 | 7 | | 43.75 | 5 | None | 1.8 |
| II | 64 | F | 10 | 4 | | 27.5 | 4 | 1 | 1.1 |
| JJ | 70 | M | 2 | 0 | | 43.75 | 5 | 1 | 3.2 |
| KK | 56 | F | 9 | 6 | | 46.25 | 6 | 6 | 1.9 |
| LL | 70 | F | 9 | 5 | | 51.25 | 5 | 2.5 | 2.0 |
| MM | 61 | F | 7 | 1 | | 35 | 10 | None | 1.2 |
| NN | 68 | M | 7 | 5 | | 33.75 | 15 | 6 | 1.1 |
| OO | 63 | F | 9 | 11 | | 55 | 57 | 35 | 1.9 |

Appendix C. Demographics and identification for bimodal cochlear-implant listeners. Note: PTA = average of pure-tone thresholds at 500, 1000, 2000, and 4000 Hz. HADS = Hospital Anxiety and Depression Scale (Zigmond & Snaith, 1983); HL = hearing loss; HA = hearing aid; RMS = root mean square

| ID | Age | Gender | HADS-Anxiety | HADS-Depression | Acoustic ear PTA | HL duration | HA use duration | RMS deviation from HA target (.5, 1, 2, 4 kHz) |
| --- | --- | --- | --- | --- | --- | --- | --- | --- |
| AAA | 60 | M | 10 | 8 | 55 | 10 | 6 | 1.6 |
| BBB | 32 | F | 8 | 4 | 68.75 | 32 | 30 | 1.6 |
| CCC | 38 | M | 10 | 4 | 88.75 | 13 | 10 | 10.0 |
| DDD | 85 | M | unknown | unknown | 68.75 | 35 | 15 | 5.1 |
| EEE | 61 | M | 6 | 9 | 70 | 20 | 10 | 6.5 |
| FFF | 76 | M | 0 | 1 | 90 | 13 | 12 | 9.8 |
| GGG | 62 | M | unknown | unknown | 76.25 | 24 | 24 | 2.7 |
| HHH | 49 | F | 5 | 5 | 100 | 5 | unknown | 11.2 |
| III | 54 | F | 2 | 2 | 56.25 | 1.5 | 1.5 | 3.0 |
| JJJ | 41 | M | 13 | 7 | 40 | 13 | 4 | 4.3 |
| KKK | 80 | M | 1 | 0 | 53.75 | 65 | 2 | 7.9 |
| LLL | 70 | M | 1 | 2 | 62.5 | 2 | 1 | 7.8 |
| MMM | 52 | F | 3 | 4 | 101.25 | 42 | 32 | 10.3 |
| NNN | 68 | M | 5 | 5 | 63.75 | 45 | 18 | 1.0 |
| OOO | 65 | F | 6 | 6 | 58.75 | 18 | 18 | 1.2 |
| PPP | 24 | F | 15 | 1 | 80 | 17 | 17 | 1.6 |
| QQQ | 25 | F | 5 | 1 | 81.25 | 21 | 21 | 7.3 |

Appendix D. CI specifications for bimodal cochlear-implant listeners listeners. Note: CI = cochlear implant

| *ID* | *CI duration* | *PTA with CI* | *CI manufacturer* | *Processor; electrode* |
| --- | --- | --- | --- | --- |
| AAA | 2 | 26.25 | Advanced Bionics | Naida CIQ70; HiRes 90K Advantage 1j |
| BBB | 1 | 26.25 | Advanced Bionics | Naida CIQ90; HiRes 90K Advantage Hi Focus Mid-Scala Electrode |
| CCC | 1 | 21.25 | Advanced Bionics | Naida CIQ80; HiFocus Mid-Scala |
| DDD | unknown | 36.25 | Advanced Bionics | Harmony processor; unknown |
| EEE | 3 | 20 | Advanced Bionics | HiRes90K; Harmony |
| FFF | 2 | 25 | Cochlear | N5 CP910 EAS processor; Nucleus Freedom CI24RE(CA) |
| GGG | 2 | 17.5 | Advanced Bionics | Naida CIQ70 with T-mic; HiRes 90K Advantage 1j |
| HHH | 5 | 16.25 | Cochlear | Nucleus CI512; N5 CP810 |
| III | 1 | 23.75 | Cochlear | Nucleus CI522 Slim Straight, CP910 |
| JJJ | 12 | 26.25 | Advanced Bionics | Naida CIQ90; HiRes 90K Mid-Scala |
| KKK | 1 | 25 | Cochlear | Nucleus 6 electrical-acoustic processor (acoustic component is disabled); Cochlear Hybrid L24 |
| LLL | 0.666667 | 20 | Cochlear | CP910; Nucleus CI512 Profile with Contour Advance |
| MMM | 8 | 21.25 | Cochlear | CP 810 NS; Nucleus 512 contour advance |
| NNN | 1 | 33.75 | Advanced Bionics | Naida CIQ90; HiRes 90K Mid-Scala |
| OOO | 2 | 18.75 | Cochlear | unknown |
| PPP | 10 | 25 | Cochlear | Cochlear CP910; unknown |
| QQQ | 3 | 21.25 | Advanced Bionics | Naida CIQ90; HiRes 90k Advantage Mid-Scala |

Appendix D. Stimulus tokens from the International Affective Digitized Sounds (IADS2; Bradley & Lang 2007) and their descriptions for each of three stimulus categories. Stimuli were obtained from stimuli developers, who offer the corpus upon request to researchers for use in an academic, non-profit, degree granting institution.

| Pleasant | | | | |  | Neutral | |  | Unpleasant | | | |
| --- | --- | --- | --- | --- | --- | --- | --- | --- | --- | --- | --- | --- |
| # | Title | | # | Title |  | # | Title |  | # | Title | # | Title |
| 110 | Baby | | 112 | Kids |  | 102 | Cat |  | 130 | Pig | 104 | Panting |
| 220 | Boy Laugh | | 151 | Robin |  | 107 | Dog |  | 242 | Female Cough | 105 | Puppy |
| 224 | Kids | | 152 | Tropical |  | 109 | Carousel |  | 243 | Couple Sneeze | 106 | Growl |
| 226 | Laugh | | 221 | Male Laugh |  | 111 | Music Box |  | 245 | Hiccup | 114 | Cattle |
| 230 | Giggling | | 378 | Doorbell |  | 113 | Cows |  | 250 | Male Sneeze | 116 | Buzzing |
| 351 | Clap | | 721 | Beer |  | 120 | Rooster |  | 251 | Nose Blow | 241 | Male Cough |
| 355 | Crowd | | 725 | Soda Fizz |  | 132 | Chickens |  | 252 | Male Snore | 244 | Man Wheeze |
| 363 | Horse Race | | 811 | Bach |  | 225 | Clap Game |  | 280 | Woman Crying | 255 | Vomit |
| 365 | Party | | 816 | Guitar |  | 254 | Video Game |  | 320 | Office | 283 | Fight |
| 366 | Casino | | 826 | Bag Pipes |  | 360 | Roller Coaster |  | 322 | Type Writer | 289 | Gun Shot |
| 601 | Colonial Music | | 802 | Native Song |  | 370 | Court Sport |  | 425 | Train | 293 | Man Sobbing |
| 808 | Bugle | | 817 | Bongos |  | 376 | Lawn-mower |  | 700 | Toilet | 295 | Couple Sobbing |
|  |  |  | |  |  | 611 | Battle Taps |  | 702 | Belch | 296 | Woman Crying |
|  |  |  | |  |  | 698 | Rain |  | 720 | Brush Teeth | 319 | Office |
|  |  |  | |  |  | 722 | Walking |  | 706 | War | 410 | Helicopter |
|  |  |  | |  |  |  |  |  | 714 | Siren | 423 | Injury |
|  |  |  | |  |  |  |  |  | 719 | Dentist Drill | 502 | Engine Failure |
|  |  |  | |  |  |  |  |  | 730 | Glass Break | 703 | Busy Signal |

­
